# Role of Mental Retardation-Associated Dystrophin-Gene Product Dp71 in Excitatory Synapse Organization, Synaptic Plasticity and Behavioral Functions

**DOI:** 10.1371/journal.pone.0006574

**Published:** 2009-08-10

**Authors:** Fatma Daoud, Aurora Candelario-Martínez, Jean-Marie Billard, Avi Avital, Malik Khelfaoui, Yael Rozenvald, Maryvonne Guegan, Dominique Mornet, Danielle Jaillard, Uri Nudel, Jamel Chelly, Dalila Martínez-Rojas, Serge Laroche, David Yaffe, Cyrille Vaillend

**Affiliations:** 1 Institut Cochin, Université Paris-Descartes, CNRS, UMR 8104, Paris, France; 2 INSERM U 567, Paris, France; 3 CINVESTAV, Physiology, Biophysics and Neurosciences, Mexico City, Mexico; 4 Centre de Psychiatrie et Neurosciences, INSERM, UMR 894, Université Paris Descartes, Faculté de Médecine René Descartes, Paris, France; 5 Department of Psychology and The Center for Psychobiological Research, Yezreel Valley College, Emek Yezreel, Israel; 6 Neurobiology, Weizmann Institute of Science, Rehovot, Israel; 7 Molecular Cell Biology, Weizmann Institute of Science, Rehovot, Israel; 8 CNRS, Neurobiologie de l'Apprentissage, de la Mémoire et de la Communication, UMR 8620, Orsay, France; 9 Univ Paris-Sud, Orsay, France; 10 INSERM, Equipe ESPRI 25 Muscle et Pathologies, Université de Montpellier I, UFR de Médecine, EA 4202, Montpellier, France; 11 Univ Paris-Sud, CNRS, UMR 8080, Centre Commun de Microscopie Electronique (CCME), Orsay, France; Hospital Vall d'Hebron, Spain

## Abstract

**Background:**

Duchenne muscular dystrophy (*DMD*) is caused by deficient expression of the cytoskeletal protein, dystrophin. One third of *DMD* patients also have mental retardation (MR), likely due to mutations preventing expression of dystrophin and other brain products of the *DMD* gene expressed from distinct internal promoters. Loss of Dp71, the major *DMD*-gene product in brain, is thought to contribute to the severity of MR; however, the specific function of Dp71 is poorly understood.

**Methodology/Principal Findings:**

Complementary approaches were used to explore the role of Dp71 in neuronal function and identify mechanisms by which Dp71 loss may impair neuronal and cognitive functions. Besides the normal expression of Dp71 in a subpopulation of astrocytes, we found that a pool of Dp71 colocalizes with synaptic proteins in cultured neurons and is expressed in synaptic subcellular fractions in adult brains. We report that Dp71-associated protein complexes interact with specialized modular scaffolds of proteins that cluster glutamate receptors and organize signaling in postsynaptic densities. We then undertook the first functional examination of the brain and cognitive alterations in the Dp71-null mice. We found that these mice display abnormal synapse organization and maturation *in vitro*, altered synapse density in the adult brain, enhanced glutamatergic transmission and reduced synaptic plasticity in CA1 hippocampus. Dp71-null mice show selective behavioral disturbances characterized by reduced exploratory and novelty-seeking behavior, mild retention deficits in inhibitory avoidance, and impairments in spatial learning and memory.

**Conclusions/Significance:**

Results suggest that Dp71 expression in neurons play a regulatory role in glutamatergic synapse organization and function, which provides a new mechanism by which inactivation of Dp71 in association with that of other *DMD*-gene products may lead to increased severity of MR.

## Introduction

Mental retardation (MR) is a clinical feature in a third of patients suffering from Duchenne muscular dystrophy (*DMD*), an X-linked genetic disease caused by mutations in the dystrophin gene [1]. The etiology of MR in *DMD* is complex and likely relies on cumulative effects of inactivation of dystrophin and other *DMD*-gene products expressed from distinct internal promoters (Dp260, Dp140, Dp116, Dp71) [2]. Full-length dystrophin (Dp427) is derived from three independent promoters that regulate its spatio-temporal expression in muscles, brain structures and cell types. Its loss in all *DMD* patients leads to muscle degeneration. At least two shorter non-muscle products, Dp140 and Dp71, that in common with dystrophin harbor the cystein-rich and carboxy-terminus domains, are also expressed in brain [1] and might be involved in MR [3]. As Dp71 is the most abundant dystrophin-gene product in adult brain [4], [5] and as *DMD* patients with mutations located in Dp71 genomic region display severe MR, Dp71 loss-of-function, mostly due to translation-terminating mutations, has emerged as a major contributing factor [2], [6].

Dp71 is expressed around brain blood vessels, most likely in perivascular astrocyte endfeet [7], and is detected in cultured astrocytes [8] and glial Müller cells in retina [9], suggesting a role for Dp71 in blood-brain barrier function. However, Dp71 mRNA has also been detected in olfactory bulb and hippocampal neurons [10], and the protein shows expression in cultured neurons [8] and in postsynaptic densities *in vivo* [11]. Yet, studies of mouse lines lacking Dp427 or all dystrophins only showed mild cognitive impairments and failed to unveil the specific contribution of Dp71 to neuronal function and to the genesis of MR [12]–[14].

Many X-linked developmental disorders associated with MR are related to proteins involved in signaling pathways that regulate cytoskeleton organization at excitatory synapses [15]. Here, to explore the specific function of Dp71, we examined its expression in neurons *in vivo* and *in vitro*, and characterized its localization in excitatory synaptic compartments. Because Dp71, as Dp427, is a key component of dystrophin-associated protein complexes (DAPC) formed by transmembrane (dystroglycans) and cytoplasmic (syntrophins, dystrobrevins) proteins that link the extracellular matrix to the actin cytoskeleton, and because DAPC may regulate signaling pathways involved in structural organization of specialized membrane-contact zones, in particular clustering of membrane receptors or ion channels [16], we characterized the Dp71-DAPC in brain and its interactions with postsynaptic proteins. Dp71-null mice [17] enabled us to examine for the first time the effects of selective Dp71-DAPC disruption on synapses organization in cultured neurons and on neurotransmission, synaptic plasticity, synapse distribution and ultrastructure in CA1 hippocampal area in the adult brain. Finally, we performed the first behavioral characterization of Dp71-null mice.

## Results

### Regional, Cellular and Subcellular Expression of Dp71 Expression in Brain

**Figure 1:**
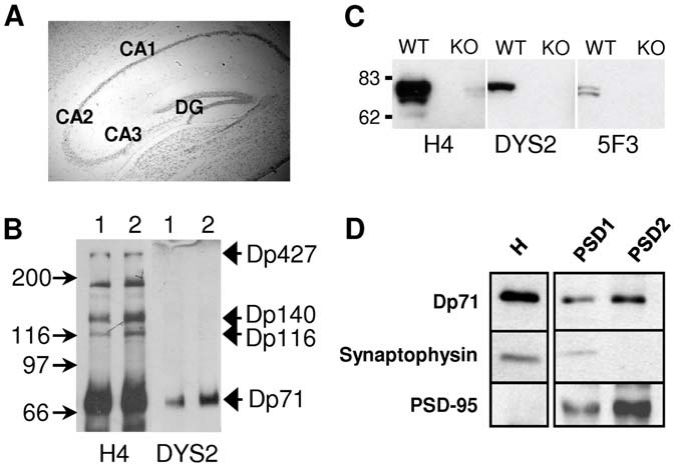
Regional and subcellular expression of Dp71 in brain. A. Hippocampal Dp71 promoter activity in CA1-3 and dentate gyrus (DG) revealed by X-gal staining in brain section from a Dp71-null mouse. B. Immunoblots showing dystrophin-gene products detected by polyclonal (H4) and monoclonal (DYS2) antibodies in dissected CA (1) and DG (2) regions of the rat hippocampal formation. Note that in addition to Dp71, the H4 antibody also revealed Dp427, Dp140 and Dp116. Molecular weight markers are indicated on the left. C. Immunoblots of hippocampal extracts from WT and Dp71-null (KO) mice. Dp71 isoforms bearing exon 78 were detected by H4 and DYS2 antibodies, and those lacking exon 78 by 5F3. The doublet of bands detected by 5F3 likely reflects presence of glycosylated and non-glycosylated forms of Dp71f [5], [21]. D. Detection of Dp71 in the postsynaptic densities. Protein extracts from subcellular fractions obtained from control mouse brains were probed with the anti-Dp71 (H4) antibody (top panel) and with the presynaptic and postsynaptic markers synaptophysin (central panel) and PSD-95 (bottom panel), respectively. H: total homogenate; PSD1/PSD2: isolated PSD fractions (Detailed distribution in subcellular fractions is shown in Fig. S3).

First, replacement of the Dp71 first and unique exon by the β-gal reporter gene in Dp71-null mice (KO) was used to estimate Dp71-promoter activity by β-gal-enzyme histochemistry (X-gal staining) on brain sections. We found that the Dp71 promoter drives high and widespread expression of the transgenic β-gal gene in both cortical and subcortical brain structures, including hippocampal subfields (Figure 1A). This confirms previous studies of Dp71 mRNA distribution in hippocampal formation [10], [18], and extends the putative expression of Dp71 to pyramidal neurons of CA1.

We then analyzed the expression of the Dp71 protein, first by immunoblotting protein extracts from adult rat and mouse brains. Using polyclonal H4 and monoclonal DYS2 antibodies, both directed against the carboxy-terminus domain common to all dystrophin-gene products, we detected the major short dystrophin-gene product of 71 kDa, Dp71, in the two main hippocampal sub-regions, the CA1 area and dentate gyrus (DG) (Fig. 1B) Transcripts encoding Dp71 are known to be alternatively spliced for exon 71–74 and/or 78 to generate multiple protein products in various tissues, which bear (Dp71d) or lack (Dp71f) specific protein-binding regions [19]. To determine whether these splice variants are expressed in hippocampus, we examined Dp71d expression with H4 and DYS2 that only react with Dp71 isoforms bearing exon 78, and Dp71f expression with the 5F3 monoclonal antibody [20], [21] that specifically recognizes variants lacking exon 78 (Figure S1, for Dp71 isoforms and antibody epitopes). As expected, all antibodies detected the 71 kDa product in WT mice (Figure 1C), indicating that Dp71d and Dp71f, the two major forms of Dp71 products resulting from alternative splice events are normally expressed in hippocampus. No expression was observed in hippocampus from KO mice, indicating that both Dp71d and Dp71f are lacking in this Dp71-null model.

We also explored expression of Dp71 using immunofluorescence on sections from mouse and rat hippocampus (Figure S2). A strong immunoreactive signal was present along the walls of blood vessels and in some astrocyte processes (Figure S2, A), which likely reflects the major expression of Dp71 in perivascular astrocyte end-feet [7]. However, both H4 and DYS2 antibodies confirmed Dp71 neuronal expression in both CA1-3 and dentate gyrus in WT and dystrophin-deficient *mdx* mice, but not in Dp71-null mice (Figure S2, B and C1-3), which is consistent with the distribution of Dp71 mRNA [10] and the X-gal staining described above. Punctate immunoreactivity corresponding to synaptic expression of Dp71 could not be clearly demonstrated on brain sections.

Western blotting of subcellular fractions of adult mouse cortex and hippocampus, validated by anti-synaptophysin and anti-PSD-95 antibodies as markers of the pre- and postsynaptic structures, was used to further detail Dp71 expression in synaptic compartments. The 71 kDa product (Dp71) was detected in total homogenate (H) and in most of the collected fractions (Figure S3), and it was clearly expressed in postsynaptic density fractions (PSD1, PSD2) (Figure 1D). The PSD2 fraction was selectively associated with expression of the postsynaptic marker, PSD-95, but not synaptophysin, confirming the presence of Dp71 in the postsynaptic densities in the adult brain.

### Dp71 is Expressed in Excitatory Synapses of Cultured Neurons

**Figure 2:**
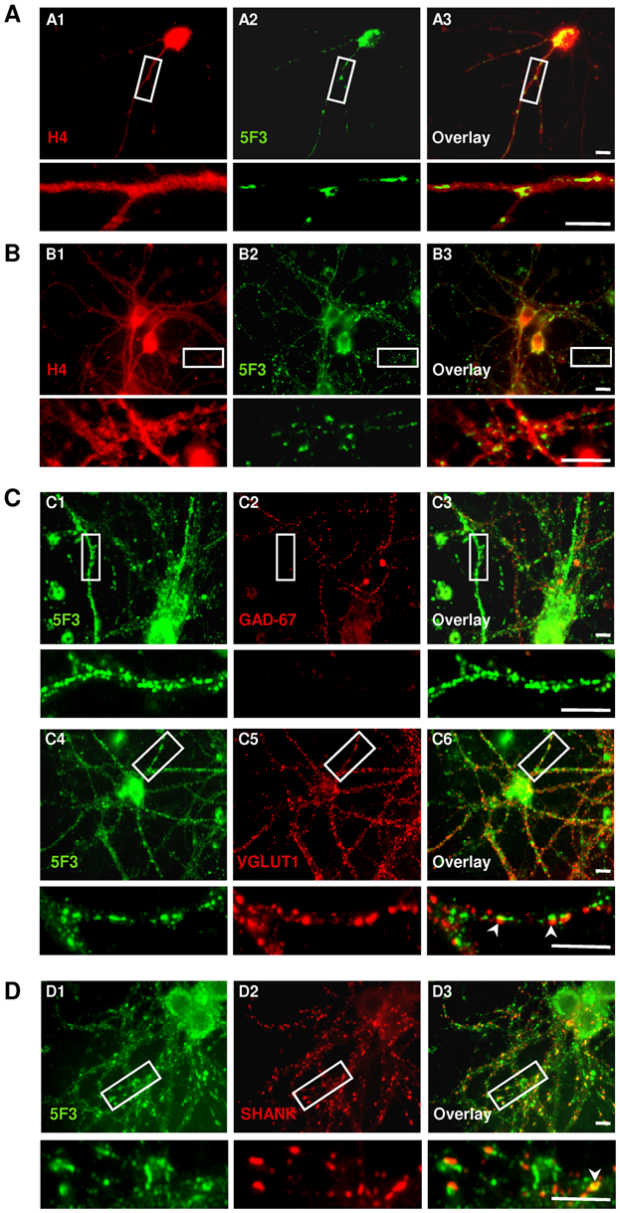
Expression of Dp71 in mouse excitatory neurons in vitro. A and B. Primary cultured neurons labeled with H4 (red) and 5F3 (green) antibodies after two (A) and 21 days (B) of differentiation. White box indicates selected neuritic segments shown below at higher magnification. Note continuous staining with H4 contrasting with discontinuous staining of 5F3 in neurites. C. Dp71 product detected with 5F3 (green, C1, C4) in subpopulations of inhibitory (C1-3) and excitatory (C4-6) neurons characterized by GAD-67 and VGLUT1 immunostaining. Overlays show co-expression of Dp71 and VGLUT1 (C6), with juxtaposition of the markers and presence of VGLUT1-positive Dp71 clusters (arrowheads), but very few co-expression with GAD-67 (C3). D. Representative images of control neurons double-labeled with 5F3 (green, D1) and the postsynaptic marker Shank (red, D2). Overlay (D3) and higher magnification of a selected dendritic segments (D3, bottom) shows juxtaposition of the two markers and the presence of Shank-positive Dp71 clusters (arrowhead). Scale bars, 10 µm.

To probe Dp71 expression in synapses of pyramidal cells and examine its putative role in synapse organization and maturation, we used immunofluorescence in primary cultured cells from mouse cortex and hippocampus. After two days *in vitro*, both H4 and 5F3 antibodies revealed immunoreactivity in the soma and along neurites in neurons from WT mice (Figure S4 A–B). H4 staining, characterized by a fine continuous, tiny and diffuse labeling (Figure 2 A1–A3), was still present in neurons from KO mice (Figure S4 C–D) and therefore did not reflect the sole expression of Dp71. In contrast, the 5F3 antibody detected specifically Dp71, as a discontinuous staining in neuritic processes of cultured pyramidal neurons from WT (Figure S4, A *vs* C), but not from KO mice. This discontinuous but strong staining revealed by 5F3 at varicosities and growth cones (Figure 2 A2, A3) was confirmed after 21 days of differentiation, when cultured cells show extensive sprouting of neuritic processes and acquisition of neuronal and synaptic phenotypes. At this stage, Dp71 had a synaptic pattern of expression, with immunoreactivity showing discontinuous granular or clustered structures along dendrites (Figure 2 B1–B3). Neuronal subpopulation expressing Dp71 was characterized by double-staining using presynaptic markers of mature excitatory (Type-1 vesicular glutamate transporter, VGLUT1) or inhibitory synapses (GAD-65/67). Neurons intensely labeled with GAD-65/67, likely corresponding to inhibitory neurons, did not express 5F3 immunoreactivity (Figure 2 C1–C3), in contrast to VGLUT1-positive excitatory neurons that clearly showed staining for Dp71f (Figure 2 C4–C6). As 24±4% of VGLUT1 clusters co-localized with Dp71 clusters, whereas only 7±3% of GAD-65/67 clusters were Dp71-positive, Dp71f isoforms thus appear comparatively more expressed in excitatory synapses.

In control neurons co-stained for Dp71 and specific synaptic markers, we found clusters of Dp71 closely in apposition to, and sometimes co-localized with both VGLUT1 (Figure 2C) and Shank (Figure 2D), another major post-synaptic density scaffolding protein interacting with glutamate receptors. Co-localization was found in 47±4% of Shank-positive clusters and in 24% of VGLUT1-positive clusters, suggesting a main localization of Dp71 in postsynaptic compartments. The percent of synapses (defined by co-localized VGLUT1 and PSD-95 clusters) positive for Dp71 was 48±4.25% and 57±4.6% of Dp71 clusters were located at synapses (n = 9 control neurons), suggesting that a significant pool of Dp71 clusters is located at mature excitatory synapses in neurons of control mice.

### The Dp71-DAPC Interacts with Synaptic Proteins

**Figure 3:**
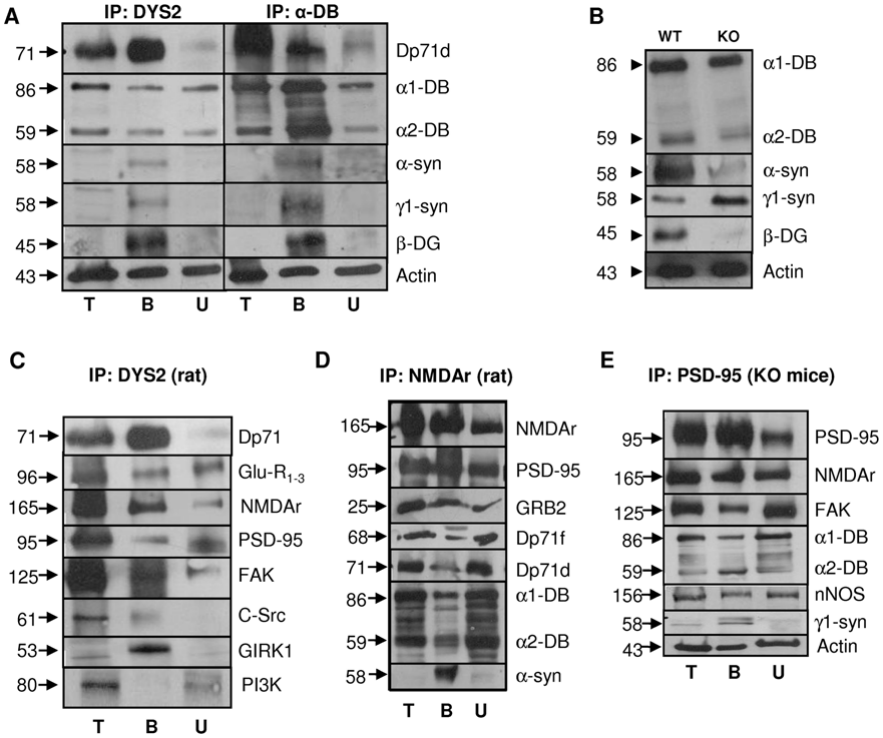
Characterization of the brain Dp71-DAPC. A. Immunoprecipitation of Dp71 with DYS2 or of αDB with αDB antibodies in rat whole-hippocampus extracts. Co-immunoprecipitated proteins were analyzed on western blots. B. Immunoblots of hippocampal extracts from WT and KO mice using antibodies directed against α-DB, α-syn, γ1-syn, β-DG and actin. C. Immunoprecipitation of Dp71 with DYS2 and westernblot analysis of interacting proteins in rat hippocampus. Co-immunoprecipitated proteins were AMPAr subunits Glu-R1-3, NMDAr subunits NR2A-B, PSD-95, FAK, C-Src and GIRK1, not PI3K. D. Immunoprecipitation using the NMDAR2A&B antibody in rat hippocampal extracts. The proteins interacting with the NMDAr complex were PSD-95, GRB2, Dp71f and Dp71d, α-DB and α-syn. E. Immunoprecipitation of the PSD-95 protein from hippocampal extracts of KO mice; NMDAr, FAK, α-DB, nNOS and γ1-syn were pulled down in the bound fraction. α-DB: α-dystrobrevins, α-syn: α-syntrophin, γ1-syn: γ1-syntrophin, β-DG: β-dystroglycan. T: Hippocampus total homogenate; B: Bound fraction; U: Unbound fraction. Proteins molecular mass (left) are indicated on the right.

To identify Dp71-associated proteins (DAPs) [11], we carried out immunoprecipitation (IP) experiments from rat hippocampal extracts using DYS2 antibody. We found that α1 and α2 dystrobrevins, α and γ1-syntrophins, β-dystroglycan and actin co-immunoprecipitate with Dp71 (Figure 3A). These results were confirmed by reciprocal IP using a monoclonal antibody directed against α-dystrobrevins. Here, Dp71 was the only dystrophin-gene product detected in the bound fraction probed with DYS2, suggesting that the other dystrophins did not take part in this specific complex.

Components of the DAPC were still expressed in brains of Dp71-null mice (Figure 3B). However, β-dystroglycan expression was drastically reduced in KO mice (∼0.5% of WT). Expression of α-syntrophins was also reduced (∼58% of WT), while γ1-syntrophin was overexpressed by more than 200%. In contrast, no difference was observed in the expression of α-dystrobrevins, suggesting that Dp71 is not required for membrane targeting of these DAPs.

Next we found that key neuronal and synaptic proteins can be co-immunoprecipitated with Dp71. These include AMPA glutamate receptor (AMPAr) subunits GluR_1–3_, the NR2A-B subunits of the NMDA subtype of glutamate receptors (NMDAr), the NMDAr-associated adapter protein, PSD-95, and the G-protein-activated inwardly rectifying potassium channel 1 (GIRK1) (Figure 3C). Two non-receptor tyrosine kinases involved in neurite outgrowth, dendritic spine morphogenesis and synaptic plasticity, the focal adhesion kinase (FAK) and C-Src, also co-immunoprecipitated with Dp71. In contrast, phosphatidylinositol-3 kinase (PI3K) was not detected in the bound fraction, suggesting absence of nonspecific binding. Other control experiments (not shown), such as IP with unspecific whole-molecule IgG followed by detection with the specific primary and secondary antibodies used in this study, and IP of protein extracts from KO mice using DYS2, did not reveal co-IP of the synaptic proteins mentioned above, which strengthens the conclusion of a specific association of Dp71, but not other dystrophin-gene products, with signaling proteins in excitatory synapses.

Reciprocal IP using anti-NR2A-B antibody allowed us to detect Dp71d and Dp71f, thus showing that both isoforms interact with the scaffold proteins associated with postsynaptic glutamate receptors in PSDs (Figure 3D). Another IP using anti-PSD-95 antibody revealed that binding of NMDAr, PSD-95, nNOS and FAK was not affected in brains from KO mice (Figure 3E), suggesting that Dp71 may be a partner, or modulator, of the core scaffold of proteins involved in the organization of glutamatergic synapses, although its congenital loss does not seem to prevent expression of these protein complexes.

### Dp71 Loss Alters Glutamatergic Synapse Maturation

**Figure 4:**
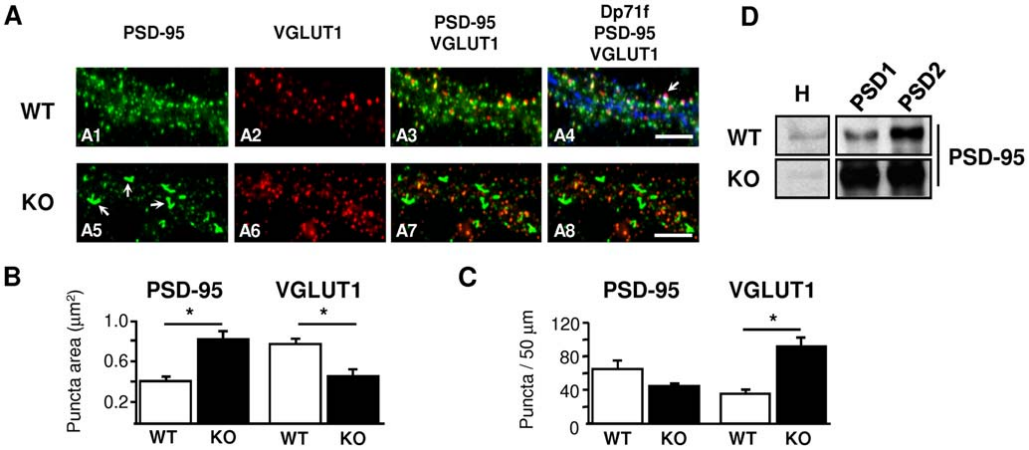
Dp71 loss alters the organization of glutamatergic synapses in vitro. A. Triple-immunofluorescence staining of dendrite segments from mature cultured neurons of WT (Top) and KO mice (Bottom), using antibodies directed against PSD-95 (green, A1, A5), VGLUT1 (red, A2, A6), and Dp71 (blue on overlay in A4). Dp71-deficient neurons show abnormally larger PSD-95 clusters on excitatory synapses (arrows in A5), a scarcity of synapses double-stained for PSD95 and VGLUT1 (A7) compared to controls (A3). Dp71 clusters positive for both PSD95 and VGLUT1 were present in WT neurons only (arrow in A4). Scale bars, 10 µm. B–C. Quantitative analyses of PSD-95 and VGLUT1 clusters. Histograms show the size of the clusters (B, puncta area) and the number of clusters per 50-µm dendritic length (C, puncta/50 µm), in WT (white) versus Dp71-null neurons (black). *p<0.001. D. Immunoblot analysis of PSD95 expression in total homogenate (H) and postsynaptic density fractions (PSD1, PSD2) from WT and KO mouse brain extracts (n = 15 mice per genotype distributed in three independent immunoblots).

In cultured neurons from Dp71-null mice, changes in the number of VGLUT1 clusters and large PSD-95 clusters were observed, suggesting that Dp71 loss may affect the clustering and distribution of these synaptic proteins. To quantify this, we used neuronal cell cultures from WT and KO mice triple immunostained with 5F3, VGLUT1 and PSD-95 (Figure 4A). In neurons lacking Dp71, the mean area of PSD-95 clusters was larger than in controls (p<0.001; Figure 4B, arrows in A5), whereas there was only a slight, non-significant reduction in the number of PSD-95 puncta (p = 0.09, NS; Figure 4C). This suggests that each cluster in Dp71-deficient neurons contains more PSD-95 protein. Using the same biochemical fractions as in Figure 1D, we compared PSD-95 expression between genotypes by autoradiography (Figure 4D). A drastic overexpression of PSD-95 was found in KO mice in the PSD1 (WT: 3232±652, KO: 10102±1021; p<0.03) and PSD2 fractions (WT: 1817±109, KO: 11025.25±802.75; p<0.01). The ratio of PSD-95 mRNA expression in KO *vs* WT mice was close to 1, as assessed by quantitative RT-PCR on whole-brain extracts (normalized to that of the housekeeping-gene, cyclophylin), indicating no quantitative variation of PSD-95 message between genotypes (not shown). This suggests that Dp71 loss selectively increases stabilization or local translation of PSD-95 [22]. We also found overexpression of PSD-93 in the PSD2 fraction (WT: 6306±537, KO: 15003±2478, p = 0.07), whereas expression of another synaptic protein, synaptophysin, was not altered, suggesting that the increased amounts of PSD-95/93 were not due to a general increase in protein expression. In all, these results suggest major alterations in the expression, organization and/or localization of PSD proteins in neurons of Dp71-null mice.

Quantification of VGLUT1 clusters revealed a different pattern of distribution (Figure 4, A2 *vs* A6), with an increased number of VGLUT1-positive puncta (p<0.001; Figure 4C) and a decrease in their size (p<0.001; Figure 4B). We then quantified the colocalization of PSD-95 clusters with VGLUT1 as an indicator of the proportion of functional synapses in cultured neurons (e.g., [23]), which was estimated 56.44±6.8% in neurons from WT mice. In neurons from Dp71-null mice, however, colocalization only reached 27.9±4.48% and the number of clusters double-stained with VGLUT1 and PSD-95 (Figure 4, A3 and A7) was significantly reduced (11.77±1.39 per 50 µm) compared to WT mice (32.62±2.22) (p<0.0001), which likely reflected a reduced density of functional synaptic junctions. Although part of PSD-95 expression in Dp71-null mice may correspond to mislocated proteins in non-synaptic zones, we however found that 62.07±5.12% of the abnormally large PSD-95 clusters (>0.4 µm^2^) observed in KO mice were VGLUT1-positive, thus indicating that accumulation of PSD-95 also largely occurs in synaptic regions. Triple-labeling for Dp71, VGLUT1 and PSD-95 (Figure 4 A4) revealed that 48±4.25% of excitatory synapses were Dp71-positive in controls, suggesting that at least half of these synapses would be affected by Dp71 loss.

### Dp71 Loss Alters Excitatory Synapse Density and Morphology in the Adult Brain

**Figure 5:**
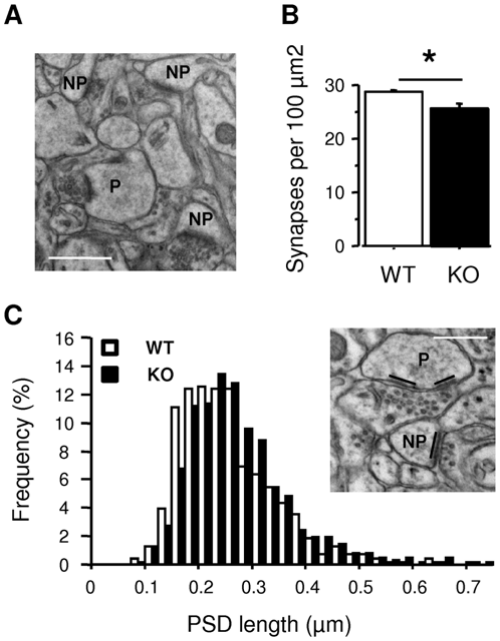
Dp71 loss affects density and morphology of axospinous asymmetric synapses in CA1 hippocampus in vivo. A. Electron micrograph showing the CA1 neuropil (stratum radiatum) with typical non-perforated (NP) and perforated (P) subtypes of excitatory synapses. Scale bar, 1 µm. B. Relative density of axospinous asymmetric synapses expressed as synapses per 100 µm^2^. *p<0.05. C. Frequency histogram distributions of the PSD length of sampled CA1 spine synapses in 25 nm bins. Insert shows measures of PSD length in NP and P synapses, with the total length of a perforated PSD estimated as the sum of the individual electron-dense traces. Scale bar: 0.5 µm.

To determine possible morphological alteration of excitatory synapses in Dp71-null mice *in vivo*, we compared axospinous asymetric synapse density and morphology in both genotypes using quantitative electron microscopy (EM). The volume of the anterodorsal hippocampus calculated from vibratome sections used for EM was comparable in WT (3.01±0.04 mm^3^) and KO mice (2.96±0.41 mm^3^) (p>0.91, not shown), suggesting no differential tissue shrinkage between genotypes. A total of 665 synapses in KO (n = 4) and 533 synapses in control mice (n = 3) were analyzed in the distal dendritic layer of CA1 anterodorsal hippocampus (Figure 5). The majority of axospinous asymetric synapses were non-perforated (92–94%), whereas perforated synapse subtype (5–7%; sample image in Fig. 5A) and multiple spine boutons (MSBs) (1–2%) were less represented. The number of axospinous excitatory synapses was reduced by **∼**11% in KO mice compared to controls (Figure 5B; p<0.029). The density of the non-perforated subtype was also reduced by **∼**13% (KO: 2.32±1.05, WT: 26.78±0.43 per 100 µm^2^; p<0.045), whereas the densities of perforated synapses and of MSBs were not affected (not shown). The distribution of PSD lengths in non-perforated synapses was slightly skewed toward the larger sizes in the KO mice (Figure 5C) (D = 0.087, p<0.024, KS test), which suggests morphological alteration of the postsynaptic active zone in Dp71-null mice.

### Hippocampal Neurotransmission and Synaptic Plasticity

**Figure 6:**
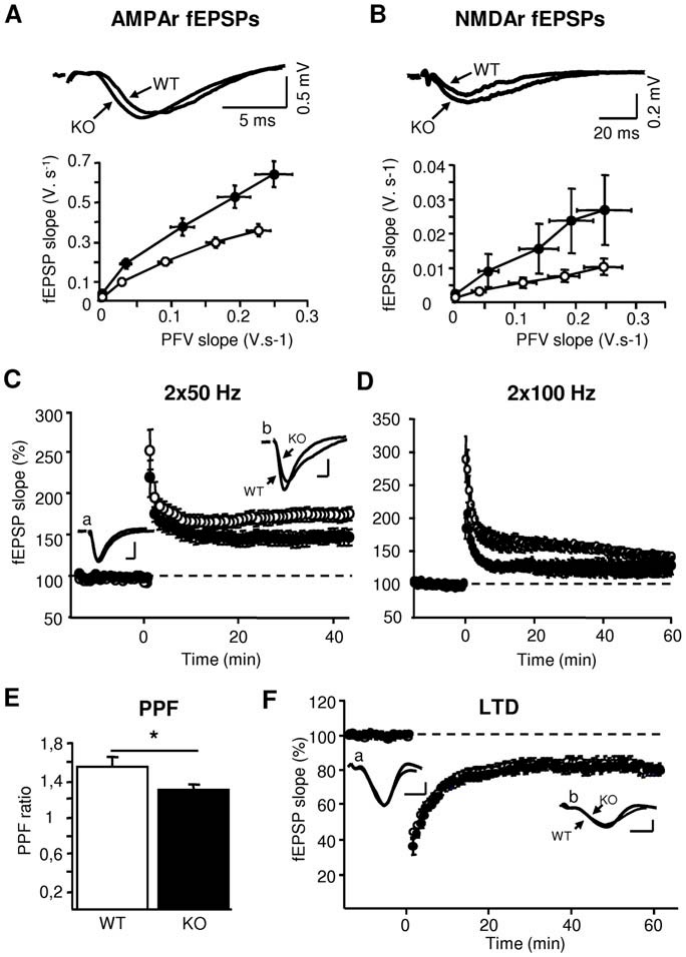
Dp71 loss alters synaptic transmission and plasticity in CA1 hippocampal area. KO: Filled symbols; WT: Open symbols. A. Basal glutamatergic transmission mediated via AMPAr. Sample traces (Top) show examples of AMPAr-mediated fEPSPs in response to single pulses (stimulation intensity: 300 µA); neurotransmission (bottom) is expressed as AMPAr-mediated fEPSP slopes plotted against presynaptic fiber volley (PFV) slopes (PFV-fEPSP coupling). B. NMDAr component of glutamatergic transmission. Sample traces of NMDAr-mediated fEPSPs (Top, stimulation intensity: 500 µA) and NMDAr-mediated PFV-fEPSP coupling (bottom). The fEPSPs were isolated in the presence of bicuculline and DNQX. C–D. Time-course of LTP induced by HFS (time 0) at 2×50 Hz (C) and 2×100 Hz (D). Synaptic strength is expressed as percent change of fEPSP slopes from baseline taken as 100% (dotted line) and plotted against time. Sample traces show averaged fEPSPs recorded in response to constant test stimuli in stratum radiatum of CA1 area before (a) and 45–60 min after induction (b). E. PPF induced by stimulus pairs (50 ms inter-pulse interval) and expressed as the ratio of the second over the first fEPSP slopes. *p<0.02.F. Time course of LTD of the fEPSP after low frequency stimulation (900 pulses at 1 Hz). Sample traces show averaged fEPSPs recorded before (a) and 60 min after induction (b) (scale bars: 5 ms, 0.5 mV).

The basic neuronal architecture of the hippocampus appeared normal in KO mice (Figure S5). Presynaptic fiber volleys (PFVs) and AMPA/kainate receptor-mediated field excitatory postsynaptic potentials (fEPSPs) were recorded in CA1 in hippocampal slices from WT (40 slices, 10 mice) and KO mice (37 slices, 9 mice). Whereas comparable PFVs were measured in KO and WT mice (p>0.5, NS; Figure S6 A), fEPSP slopes were significantly higher in KO mice (p<0.001; Figure S6 B), resulting in enhanced coupling between PFV and fEPSPs (Figure 6A), i.e., enhanced synaptic transmission. When the NMDAr component of glutamatergic transmission was isolated (6 slices from 3 mice of each genotype), no significant group difference was found in PFVs' slopes (p>0.7, Figure S6 C), but NMDAr-mediated fEPSPs (Figure S6 D) and PFV-fEPSP coupling (Figure 6B) were again significantly enhanced in mutant mice (p<0.05). Analyzing PFVs in the absence of EPSPs (with 10 µM bicuculline, 10 µM DNQX, and 30 µM APV in bathing medium) showed no difference between KO (n = 6) and WT mice (n = 5) (PFV slope: p>0.8; PFV amplitude: p>0.8, data not shown), which confirms that the increased neurotransmission in mutants was not due to changes in afferent fiber density or excitability.

We then examined long-term potentiation (LTP) using two protocols of high-frequency stimulation (HFS, 2×50 Hz, 2×100 Hz). Stimulus intensities were adjusted to obtain comparable fEPSP slopes in the two genotypes before the induction of LTP (WT: 0.107±0.01 V.s^−1^, KO: 0.110±0.001 V.s^−1^, p>0.5). When induced by two trains at 50 Hz (WT: 12 slices, 6 mice; KO: 8 slices; 5 mice, Figure 6C), the level of LTP was comparable between genotypes during the first 15 min after HFS, but the magnitude of LTP expressed after 30 min was significantly lower in KO than in WT mice (last 15 min, p<0.04). When LTP was induced by a stronger HFS (2×100 Hz; WT: 11 slices; 6 mice; KO: 11 slices, 5 mice, Figure 6D), the magnitude of LTP was significantly reduced in KO mice (128.40±1% of baseline) compared to WT mice (160.18±1.04%) during 1 h post-HFS (p<0.02). Immediately post-tetanus (30 s), fEPSP potentiation was already significantly smaller in KO (185.08±15.88%) than in WT mice (289.26±34.64%) (p<0.02), and remained significantly reduced for at least 30 min (p<0.01). During the last 10 min of recording, LTP levels were comparable in KO (125.16±2.76%) and WT mice (143.68±1.25%) (p>0.2), indicating that this stronger HFS protocol induced stable and comparable LTP maintenance in the two genotypes.

We also analyzed forms of short-term plasticity, paired-pulse facilitation (PPF) and posttetanic potentiation (PTP). The slopes of the first fEPSP were adjusted to similar levels in KO (n = 5, 14 slices) and WT mice (n = 5, 12 slices) before induction of PPF (p>0.6). The PPF ratio was however significantly decreased in slices from KO compared to WT mice (p<0.02, Fig. 6E), suggesting alteration in presynaptic mechanisms of glutamate release. PTP was induced by delivering HFS (2×100 Hz) in the presence of 80 µM APV. The transient PTP, which decayed to baseline within a few minutes in both KO (8 slices, 5 mice) and WT mice (7 slices, 5 mice), was statistically comparable between genotypes (Figure S6 E). This confirmed that LTP in both genotypes is NMDAr-dependent and suggests that LTP impairment in Dp71-null mice is not due to reduced presynaptic efficiency.

Finally, we examined LTD induced by LFS (900 pulses at 1 Hz) in area CA1 in 9 slices from WT (n = 5) and 8 slices from KO mice (n = 4). The time course and magnitude of LTD were comparable between genotypes during 1 h post-LFS (p>0.8; Figure 6F), suggesting that reduced LTP in KO mice is not due to facilitation of LTD mechanisms.

### Behavioral Analysis of Dp71-Null Mice

#### Motor Capacities

**Figure 7:**
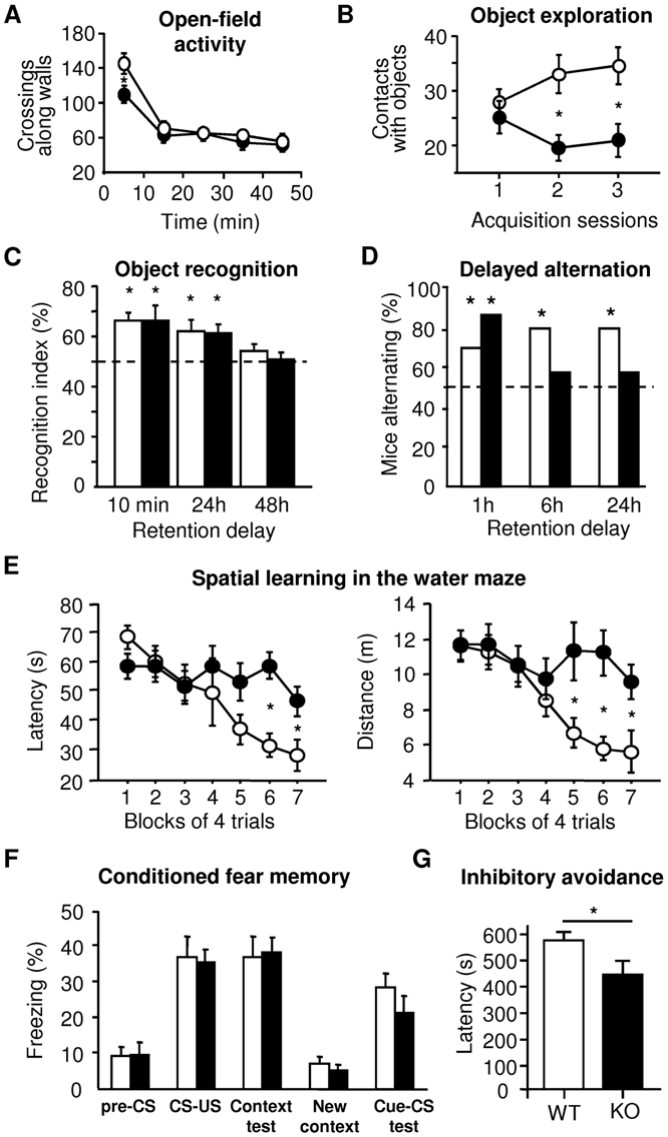
Selective alterations of exploratory behavior and learning and memory performance in Dp71-null mice. KO: Filled symbols; WT: Open symbols. A. Spontaneous locomotor activity during exploration of an empty open-field (9 KO, 10 WT). The number of squares crossed along the walls for 50 min is shown by bins of 5 min recorded every 10 min. *p<0.02. B. Object investigations expressed as the number of contacts made with three different sets of objects used during successive acquisition sessions in the object recognition task (13 KO, 16 WT). *p<0.01. C. Object-recognition memory performance at retention delays of 10 min, 24 h and 48 h. *significantly different from chance (50%), p<0.05. D. Spatial alternation in a T-maze. Data are % of mice alternating (14 KO, 24 WT) at retention delays of 1, 6 and 24 h. *significantly different from 50%, p<0.05. E. Spatial learning in the water-maze (12 KO, 10 WT). Escape latencies (left panel) and distance swum to reach the platform (right panel) monitored over 7 days (1 block of 4 trials per day). *p<0.05. F. Contextual and cued-fear memory. Graph shows percent freezing (12 KO, 13 WT) before (pre-CS) and immediately after administration of the unconditioned stimulus (US, footshock) during learning (CS-US), and during retention trials 24 h later in the same context (context test), or in a new context before (new context) and after presentation of the auditory CS (cue-CS test). G. Inhibitory avoidance learning (17 Dp71-null, 12 WT). Retention performance at 24 h is expressed as the latency to step into the electrified zone. *p<0.05.

KO and WT mice showed comparable fall latencies in the inverted grid test (44.25±4.53 s and 41.38±4.82 s, respectively) and comparable latencies to grip the wire in the suspension test (2.54±0.43 s and 2.36±0.49 s) (p>0.6). Although KO mice had reduced locomotor activity during the first 10 min of open-field exploration (p<0.05), they displayed fast habituation and similar baseline locomotion compared to controls from approximately 15 min until the end of testing (Figure 7A). KO mice also showed normal avoidance of the center zone of the arena compared to controls (not shown). Both genotypes showed comparable locomotor activity in a cross-maze (number of arms visited; KO: 19.44±1.64; WT: 22.05±1.73; p>0.3) and similar rearing frequency in both experiments (not shown). This indicates that basal motor functions were not affected by the loss of Dp71, in agreement with the lack of overt muscle histopathology in this model (not shown) and the absence of Dp71 expression in differentiated skeletal muscles [17].

#### Exploration and Emotional Reactivity

The initial reduction of activity shown by KO mice during forced exploration of the open-field was confirmed when mice were repeatedly exposed to the open-field during habituation sessions preceding object recognition testing (number of squares crossed, p<0.002; crossings in central area, p<0.05; rearings, p<0.05). A clear reduction of object exploration was also detected during both acquisition and retention sessions of this task: Object investigations were comparable between genotypes on the first acquisition session, whereas they were significantly reduced in KO mice when exposed to new sets of objects on successive sessions (Figure 7B). A significant decrease in the number of contacts with objects (p<0.007) and in the duration of object exploration (p<0.03) was observed during both acquisition and retention. In contrast, no reduction of exploratory behavior was observed when KO mice were exposed to a novel arena that offered a retreat opportunity into a small familiar home-box (free-choice exploration, all parameters p>0.5, not shown). Finally, no difference was found between genotypes in the plus-maze anxiety test (% entries in open arms: WT: 16.61±3.82; KO: 13.14±4.94, p>0.2). Thus, KO mice exhibited reduced exploratory and novelty-seeking behaviors, which could not be directly attributed to enhanced emotional reactivity.

#### Learning and Memory

In the object recognition task, the percent time spent exploring each of the two objects during acquisition was comparable in WT (49.3 and 50.69±1.89%) and KO mice (50.69 and 49.31±2.74%). Following a 10 min, 24 h or 48 h delay, one of the familiar objects was replaced by a novel object and the time spent exploring the novel object (*recognition index*) was measured. No statistical difference was detected between groups: The recognition index differed significantly from chance in both WT and KO mice at the 10 min and 24 h delays, but not at the 48 h delay (Figure 7C), both genotypes showing good and comparable short and long-term recognition memory, and a similar rate of forgetting.

In the cross-maze, mice of both genotypes showed a comparable number of different arm choices within every set of 4 arm entries (WT: 3.28±0.04; KO: 3.19±0.06; p>0.3) and their performance was not affected by stereotyped response patterns (% chaining: WT: 19.99±3.65; KO: 24.37±5.05, p>0.4), suggesting that spontaneous alternation is not impaired in KO mice. In contrast, KO mice showed impaired performance when spatial delayed alternation was assessed in a T-maze (Figure 7D). Only WT mice showed significant alternation rates at 6 h and 24 h, suggesting impaired spatial memory performance at long delays in KO mice.

During spatial learning in the water-maze, acquisition of the navigation task was clearly impaired in Dp71-null mice, which, in contrast to WT mice, showed no significant improvement of performance over training (Figure 7E), as expressed by the latency (last 4 blocks of trials, genotype effect: p<0.01) and distance swum (p<0.01) to find the platform. While mice of both genotypes had comparable swim speed and thigmotaxis (both parameters p>0.2), KO mice displayed higher accelerations during progression segments (p<0.05) and a high rate of 360°-rotation of the animal's body (p<0.0001), suggesting darting or impulsive-like responses and increased path tortuosity. When training was performed in a smaller pool with a larger platform and with one more trial per day (see Materials and Methods S1), conditions that likely reduce task difficulty, WT mice showed faster learning (Figure S7), but KO mice were still impaired, showing delayed acquisition of the task (Escape latency: p<0.05; day 2: p<0.008). In these conditions, however, their performance progressively improved and reached similar scores as that of the controls on the last two training days (p>0.2). The KO mice also displayed slightly longer latencies during a reversal test (p>0.05, NS), which was reminiscent of the performance deficit during acquisition.

Associative learning was assessed using three different fear-conditioning protocols: The hippocampus-dependent contextual and amygdala-dependent cued-fear conditioning, and inhibitory avoidance. Retention performance was evaluated 24 h post-training by measuring freezing to the context alone (context test) and to the auditory conditioned stimulus (CS, tone) delivered in a new context (cue-CS test), and by the latency to enter the electrified zone in inhibitory avoidance. KO and WT mice expressed comparable freezing behavior during habituation, acquisition and retention of both contextual and auditory-cued fear conditioning (Figure 7F). Both groups also displayed comparable performance during acquisition of inhibitory avoidance learning (not shown), but the latency to enter the electrified zone was shorter in KO than in WT mice at the 24 h retention delay (Fig. 7G).

## Discussion

In *DMD*, Dp71 deficiency is associated with MR in a subpopulation of patients [6] and is thought to contribute to the severity of MR phenotypes. Dp71 function is however unclear and complex, as the protein appears to be expressed in both perivascular astrocyte end-feet and neurons. Here, we further characterized this complex pattern of expression and show that Dp71 interacts with specialized modular scaffolds that organize signaling complexes at glutamatergic synapses. We also found that Dp71 loss affects synaptic maturation and function, and results in deficits in selective cognitive functions.

The data provide evidence for an association of Dp71-DAPC with the multi-protein scaffolds that cluster glutamate receptors and organize signaling proteins required for synaptic transmission and plasticity, including NMDAr and AMPAr subunits, and other PDZ-containing proteins such as PSD-95 and nNOS. This specific Dp71-DAPC contains the two main splice variants, Dp71d and Dp71f, along with β-dystroglycan, α- and γ1-syntrophins and α-dystrobrevins. Dp71-DAPC is also associated with key signaling proteins such as FAK, c-Src and Grb2, proteins known to contribute to neurite outgrowth, dendritic spine morphogenesis and synaptic plasticity [24]–[26]. It has been shown that in the nervous system DAPC binds nNOS [27], ErbB4 neuregulin receptors [28] and transmembrane channels [29], [30] through the PDZ domain of syntrophins, whereas the adaptor protein Grb2 mediates FAK/β-dystroglycan interactions [31]. Future studies will determine whether such mechanisms can explain how Dp71-DAPC binds to synaptic proteins. Interestingly, we found that Dp71-DAPC also interacts with GIRK1 (Kir3.1) potassium channels typically detected in perisynaptic lipid rafts [32]. This suggests cross-linkage between organizing molecules of the PSD and lipid rafts and the possible existence of two distinct pools of Dp71-DAPC in PSD and in perisynaptic regions. In brains of Dp71-null mice, loss of Dp71 resulted in reduced levels of β-dystroglycan and α-syntrophin. This, however, did not prevent the formation of NMDAr complexes, which also maintained stable associations with α-dystrobrevins and α-syntrophin, suggesting that distinct mechanisms are involved in postsynaptic membrane targeting of Dp71, DAPs and members of glutamate-receptor complexes.

Emerging notion conceptualize DAPC as a scaffold for proteins involved in membrane stabilization and transmembrane signaling [16]. DAPC stabilizes large clusters of acetylcholine receptors at the neuromuscular junction in association with utrophin [33] and modulates GABA_A_-receptor clustering in association with Dp427 in central inhibitory synapses [34] without being required for membrane anchoring of these receptors. Here, we found that cultured primary neurons from Dp71-null mice have abnormally large clusters of PSD-95 protein and a reduced number of mature synapses. Hence, whereas Dp71-DAPC seems dispensable for postsynaptic targeting of glutamate receptor complexes, it may be a key modulator of their organized distribution. At the ultrastructural level in the adult brain, we found that KO mice have a reduced number of excitatory synapses (∼11–13%) with larger cross-sectional PSD length, which parallels our findings in cultured neurons and suggests reduced synapse density and altered morphology of the postsynaptic active zone in Dp71-null mice.

Using the hippocampal slice preparation, we found that both AMPAr and NMDAr-mediated components of CA1 glutamatergic transmission were drastically enhanced in KO mice. This was associated with reduced LTP levels, yet larger magnitude of LTP could be achieved in KO mice with stronger tetanization protocols, whereas LTD was not affected. Given the range of proteins found to interact with Dp71-DAPC, such alterations could be attributed to several pre- and postsynaptic mechanisms. A presynaptic hypothesis involving increased glutamate release is suggested by the overall increase in neurotransmission and reduced paired-pulse facilitation observed in KO mice. Moreover, in cultured neurons congenital loss of Dp71 was associated with an altered distribution of the presynaptic vesicular transporter VGLUT1. A purely presynaptic account seems however unlikely as PTP under NMDAr blockade was comparable between genotypes. On the other hand, the contribution of postsynaptic mechanisms to enhanced synaptic transmission in Dp71-KO is suggested by the increased PSD length, which may correlate with aggregation of receptors and signaling molecules at the active zone [35]–[37], and by the abnormal accumulation of PSD-95 in Dp71-deficient synapses, which could increase NMDAr gating [38], promote the delivery of AMPAr to synapses and increase AMPAr currents [39]–[41]. Such changes could explain that LTP expression was partially occluded in the Dp71 mutant mice, as suggested in mice overexpressing PSD-95 [40], [41]. At this stage, however, the primary mechanisms linking Dp71 loss to altered synaptic function remain uncertain. The observed synaptic alterations indicate that the congenital loss of Dp71 significantly impinges synapse organization and maturation, but does not imply that Dp71 takes part actively to experience-driven synaptic plasticity in the adult brain. Moreover, other factors related to Dp71 function in other cell types might also contribute to the phenotype: The Dp71-DAPC appears to endorse a major role in the clustering of aquaporin (AQP4) channels in perivascular astrocytes [7] and its disruption may alter the blood-brain barrier and water and K+homeostasis, which could indirectly affect neuronal function [42].

The diversity of mechanisms presumably modified as a consequence of Dp71 loss underscores the complex cellular and subcellular distribution of Dp71 in brain. The finding that a pool of Dp71 may act as a regulator of glutamatergic synapse maturation, architecture and function provides valuable insights into identifying some of the complex functional pathways underlying MR in *DMD*. Special interest should focus on factors and signals that coordinate the interplay between the pre- and postsynaptic compartments during synapse morphogenesis and plasticity, which appear involved in several types of mental disorders [15]. The behavioral characterization of Dp71-null mice revealed selective disturbances characterized by reduced exploratory behavior, slight retention deficits in inhibitory avoidance, and impaired spatial learning and memory. In Dp71-null mice, exploration deficits may partly relate to emotional reactivity, neglect and/or withdrawal from novelty-seeking behavior; disorders that have been associated with enhanced glutamatergic transmission [43]. Such deficiencies that may compromise the patients' autonomy and social rehabilitation have not as yet been systematically assessed in *DMD* (but see [44], [45]). Impaired navigation in the water maze and spatial alternation in the T-maze suggest an alteration in hippocampus-dependent encoding and/or consolidation of spatial information, in line with the synaptic and neurophysiological alterations observed in Dp71-null mice in this structure. However, the ability of Dp71-null mice to reach a normal level of performance in the navigation task when task difficulty is reduced, the mild deficit in inhibitory avoidance and unaffected retention performance in object recognition and contextual fear conditioning suggest that certain hippocampal functions are preserved. Although the Dp71-null mouse constitutes a unique model to explore the specific contribution of congenital Dp71 loss in brain, it is important to note that the genesis of MR in *DMD* likely encompass cumulative inactivation of Dp427, Dp71 and Dp140 [2], [3]. The function of Dp140 in brain is at present unknown [1]. Loss of Dp427 in *mdx* mice alters GABA_A_-receptor clustering [34], which has been associated with abnormal enhancement of hippocampal LTP [13], [46], reorganization of inhibitory synapses and morphological alterations of hippocampal excitatory-synapse subtypes [47], and behavioral impairments mainly characterized by mild memory retention deficits [13], [48]. Here we show that Dp71, in addition to its potential role in the control of the blood-brain barrier in relation to its expression in perivascular astrocytes [7], plays a role in glutamatergic synapse organization and function and that its loss also results in cognitive impairments. The present results suggest stronger learning impairments in Dp71-null mice than in *mdx* mice, which supports the hypothesis that Dp71 loss has a major role in the genesis of cognitive deficits in DMD. However, the two models display distinct synaptic and behavioral alterations and it would thus be interesting in future studies to examine the consequences of mutations preventing the expression of both proteins. This could be tentatively addressed with the mdx^3CV^ mutant mouse that lacks all dystrophin-gene products, but the few studies to date show that this model does not replicate the behavioral and neurophysiological alterations reported in *mdx* [12], [14] and Dp71-null mice. The possibility that residual expression or compensatory mechanisms may attenuate the loss of dystrophins in this model has not been resolved. The generation of new mouse models with brain-specific mutations and combined loss-of-function of several dystrophin-gene products will thus be an important step to determine the respective involvement of GABAergic and glutamatergic functions in the neural basis of MR in *DMD*.

## Materials and Methods

### Ethics Statement

Animal studies complied with the European Communities Council Directive (CEE 86/609) for animal care and experimentation and were conducted following the guidelines of the animal facilities in Orsay (France) approved by the national direction of veterinary services (Direction des Services Vétérinaires, DSV-France, agreement # B91-429 for the rats and B91-471-104 for the mice).

### Animals

Male Wistar rats, dystrophin-deficient *mdx* mice and Dp71-null mice (KO) and their control WT littermates (>3 mo old) were used. Rats were purchased from Charles River (France); *mdx* mice and their controls of the C57BL/10ScSn strain were originally purchased from Jackson Laboratories (USA) and Harlan (France), respectively, and then bred in the mouse transgenic facility in Orsay (France) as described [13]; Dp71-null and littermate controls were produced in the animal facility at Weizmann Institute (Israel). Animals were kept under a 12-h light-dark cycle (light on: 7.00 a.m.) with food and water *ad libitum*. Generation of Dp71-null mice by homologous recombination has been described previously [17]. Briefly, most of the first and unique exon and a small part of the first intron of Dp71 have been replaced by the promoter-less gene encoding a β-gal-neomycin resistance chimeric protein, which specifically abolished the expression of Dp71 without interfering with the expression of other *DMD*-gene products. Females heterozygous for the mutation were backcrossed for at least 9–10 generations to balb/c males to generate the KO and littermate WT mice. Genotype was determined by PCR analysis of tail DNA. Functional analyses were conducted blind to the genotype.

### Biochemistry

#### Antibodies

The polyclonal H4 antibody directed against the C-terminal part of dystrophins and recognizing Dp71, the specific monoclonal 5F3 antibody directed against the last 31 residues of Dp71 isoforms lacking exon 78 (Figure S1), and the antibodies directed against β-dystroglycan, α- and γ1-syntrophins were all generated by D. Mornet and characterized in detail. The monoclonal DYS2 antibody (Novocastra Laboratories, Newcastle, U.K.) is directed against the C-terminal part of dystrophins, in the same region of the protein as H4. All antibodies used and sources are described in Table S1. Fluorescein- or Cyanine 3-conjugated goat anti-rabbit, donkey anti-rabbit and goat anti-mouse secondary antibodies were from Jackson ImmunoResearch (West Grove, PA, USA).

#### Western blot analyses

Brains were removed immediately after decapitation and whole hippocampi were dissected out. In some experiments the dentate gyrus and CA1-3 hippocampal subfield were dissected and analyzed as separate samples. Mouse dissected brain structures were homogenized with a polytron (30 s, 4°C, full speed) in 5 volumes (wt/vol) of extraction buffer. Protein concentration was evaluated by nanodrop. Equivalent amounts of protein were resolved on 10% (rat) or 8% (mouse) SDS-PAGE and electrotransferred to a nitrocellulose membrane.

For the subcellular and PSD fractionation, five mouse brains were used in a procedure adapted from Huttner et al. [49]. On the last step of the protocol, the PSD fractions PSD1 and PSD2 were resuspended as described elsewhere [50], [51] (see Materials and Methods S1 and Figure S3 for a detailed description).

For immunoprecipitation assays, one mg-protein of rat hippocampus was homogenized in RIPA 1 buffer (50 mM Tris-HCl pH 8, 150 mM NaCl, 1% Triton X-100, 0.1% SDS, 1% sodium deoxycholate, and 2.5 mM EDTA) containing protease inhibitors and incubated overnight at 4°C with appropriate antibody. Protein-antibody complexes were mixed with Protein A/G Plus Agarose (Santa Cruz Biotechnology, USA) and incubated overnight at 4°C. After extensive washing with buffer RIPA 2 (50 mM Tris-HCl pH 8, 150 mM NaCl, 0.2% Triton X-100, 0.02% SDS and 0.2% sodium deoxycholate) the resin containing the protein-antibody complexes was suspended in sample buffer (1/3 of initial volume), boiled and analyzed by western blot (bound fraction). The buffer of the crude extract used for immunoprecipitation was diluted 1:10 and analyzed in parallel (unbound fraction).

#### Cell culture

Cortex from individual mice at embryonic day 15.5 (E15.5) were dissected, dissociated by trypsin treatment and mechanical trituration, and plated onto polylysine-coated glass coverslips (14 mm in diameter) (SIGMA-Aldrich) at a density of ∼5000 neurons/cm^2^. Cultures were maintained in neurobasal medium complemented with B27 and glutamate (Invitrogen) as described elsewhere [52].

#### Immunocytochemistry

Cultured cells were fixed with 100% methanol at −20°C for 10 min, permeabilized with 0.1% triton X-100, blocked in PBS plus 3% BSA, and then incubated with appropriate primary and secondary antibodies. For triple-staining assays, Zenon labeling technology (Molecular probes, Invitrogen) was applied to enable the use of two monoclonal antibodies on mouse tissues. For quantification of immunostained puncta, images were taken with a 40X oil-objective, automatically threshold and analyzed with the ImageJ software (NIH, USA, http://rsb.info.nih.gov/ij/). A total of 35 primary dendrites from 7 neurons were analyzed for each genotype. For each dendrite, a 50-µm segment was analyzed (250 µm per neuron). Each experiment was repeated at least twice with independent neuronal cultures. Neurons from each genotype were analyzed using identical imaging conditions to quantify Dp71 co-localization with presynaptic (VGLUT1) and postsynaptic (SHANK) markers of mature excitatory synapses and with a presynaptic marker of inhibitory synapses (GAD-65/67). A synapse was defined by juxtaposition of the pre- and postsynaptic markers.

#### Immunohistochemistry

Fresh frozen brains were processed as described [53]. Sections were fixed for 3 min in acetone/methanol (1:1) at −20°C, stained for 24 h at 4°C with primary antibody and then incubated 45 min with secondary antibodies. In some experiments the tissue was fixed by transcardiac perfusion with 4% paraformaldehyde in 0.1M-phosphate buffer (pH 7.4). Controls were prepared by omitting the primary antibody; in these controls, no specific staining could be detected.

#### X-gal staining

Beta-galactosidase enzyme histochemistry was performed on both fresh-frozen and formalin-fixed brains from Dp71-null mice. Sections were washed in 0.1M PBS, stained for 1 h at 40°C with X-gal (Promega France), dehydrated and then cleared in Xylene. Dimethyl Formamide (0.4%), MgCl_2_ (1M), potassium ferrocyanide (5%) and ferricyanide (5%) were added to the staining buffer.

### Electron microscopy

Brain tissue was processed as previously described [47] (see Materials and Methods S1). Ultrathin sections (70-nm thick) were visualized at ×14000 using a Philips EM208 transmission electron microscope (Philips Electron Optics, Eindhoven, NL) and photographed with a CCD AMT digital camera (Hamamatsu, Japan). Synapses were examined in the distal stratum radiatum (∼150 µm from the CA1 pyramidal cell bodies). Axospinous symmetric (excitatory) synapses were identified by a thick PSD, synaptic cleft and presynaptic component with synaptic vesicles [54]. Those with one or more perforations in the PSD were classified as perforated and those showing a continuous PSD as non-perforated (Figure 5A). Single presynaptic terminals contacting more than one postsynaptic spine were defined as multiple spine boutons (MSBs). The relative density of asymmetric synapses (synapses per µm^2^) and the cross-sectional length of their PSDs were estimated from 15 counting frames per animal (total sampled neuropil area: 663.33 µm^2^). When the PSD was perforated, the sum of the individual trace lengths was considered for PSD length (Figure 5C, insert) [36].

### Electrophysiology

Acute hippocampal slices from mice (3–4 mo) were used as previously described [13]. Extracellular synaptic responses were recorded in CA1 stratum radiatum to monitor PFVs and fEPSPs evoked by electrical stimulation of the Schaffer collateral/commissural fibers at 0.1 Hz. Input/output curves were constructed by measuring the slopes of fEPSPs mediated by AMPAr and NMDAr at different stimulus intensities (100–500 µA; 60 µs duration, averages of 3 responses). NMDAr-mediated responses were isolated in the presence of the GABA_A_ receptor antagonist, bicuculline (10 µM), and the AMPA/kainate receptor antagonist 6,7-Dinitroquinoxaline-2,3-dione (DNQX, 10 µM). Addition of D-APV (30 µM) was used to analyze PFVs in the absence of EPSPs. PPF was induced by stimulation with pairs of pulses with a 50-ms interstimulus interval and quantified as the ratio of the second over the first fEPSP slopes. To investigate LTP, the initial slope of 3 averaged fEPSPs was measured for 15 min (baseline) and tetanic stimulation consisted of two trains, 20 s apart, of HFS of either 50 Hz or 100 Hz (duration: 1 s at test intensity). LTD was induced by low-frequency stimulation (900 pulses at 1 Hz). Testing with single pulses was resumed for 60 min after induction of synaptic plasticity.

### Behavioral testing

#### Subjects

Eighty-six male mice on a main balb/c background (40 KO and 46 WT littermates), aged 3 to 8 month, were used. Because mice with inbred balb/c background are considered poor learners in spatial tasks, outcrosses between female balb/c-heterozygous for Dp71 and C57BL/6 male mice were undertaken to obtain CB6F1 hybrids tested in the water maze. The behavioral paradigms have been conducted as described [13], [48], [55], [56] with few adaptations. Detailed protocols are provided as supporting information in Materials and Methods S1.

### Statistical analyses

Data are reported as means±SEM. They were analyzed using ANOVA with genotype as the between-subject factor, complemented by a within-subject factor analysis for time or place dependence of effects (Statview 5.0). One set of data, the latencies recorded in inhibitory avoidance, did not pass the normality and equal-variance tests (SigmaStat 2.0, SPSS Inc.) and was therefore analyzed with non-parametric Mann-Whitney U test. In object recognition, comparison of recognition indexes to chance was performed using univariate *t*-tests. In delayed alternation, a chi-square analysis was used to compare alternation rates to chance. The distribution of PSD length in the EM study was analyzed with the Kolmogorov-Smirnov (KS) test.

## Supporting Information

Figure S1 Schematic representation of Dp71 isoforms. (A) Dp71 is transcribed from a promoter located between exons 62 and 63 of the DMD gene; it has a unique N-terminus of seven amino acids and contains the cysteine-rich and common C-terminal domain of dystrophins, a region for binding to DAPs complexes. (B) Dp71 alternative spliced transcripts. Dp71d isoform lacks exon 71 and Dp71f isoform lacks exons 71 and 78 (Marquez et al., Neuroscience 118:957–966, 2003). Deletion of exon 78 changes the reading frame, resulting in the replacement of the last 13 hydrophilic amino acids of dystrophin with 31 new hydrophobic amino acids in the Dp71 protein. Variants of Dp71 protein with minor expression in brain have also been described (Austin et al., Neuromuscul Disord 10(3):187–93, 2000): Dp71Δ110m is derived from Dp71 transcripts deleted for exons 71–74 and contains the C-terminal sequence of muscle dystrophin, while Dp71Δ110a contains the alternative 31-amino acid C-terminal sequence due to the splicing of exon 78 (Austin et al., J Biol Chem 277:47106–47113, 2002). The location of the epitopes recognized by antibodies H4, DYS2 and 5F3 are indicated.
(1.01 MB TIF)

Figure S2 Immunofluorescence detection of Dp71 in brain sections. Hippocampal expression of Dp71 isoforms revealed by H4 on rat brain sections. Representative images show Dp71 expression in (A) walls of blood vessels and perivascular astrocytes (arrowhead), and (B) in the granule-cell layer (gcl) of dentate gyrus as incomplete rings of immunoreactivity circling granule cell bodies and small to large dots around or within cell bodies. C. Immunolabeling of Dp71 with H4 antibody in the gcl and walls of blood vessels (arrowheads) in WT (C1), mdx (C2) and Dp71-null mice (C3).
(2.61 MB TIF)

Figure S3 Fractionation study. A. Schematic representation of the fractionation protocol (detailed description in Materials and Methods S1). B. Protein extracts from subcellular fractions obtained from control mouse brains probed with the anti-Dp71 (H4) antibody (top panel). Bottom panels show expression of the presynaptic and postsynaptic markers synaptophysin and PSD-95, respectively. Fractions as follows: H, total homogenate; S1, cell soma; P1, dense nuclei-associated membrane; S2, supernatant, P2: crude synaptosomal membrane, S3, cytosolic; P3, light membrane compartment; LS1, soluble; LP1, synaptosomal membrane; LP2, synaptic-vesicle enriched; SPM, synaptic plasma membrane; PSD1/PSD2, postsynaptic density fractions.
(1.84 MB TIF)

Figure S4 Expression of Dp71 in cultured neurons of control (WT) and Dp71-null (KO) mice. Immunofluorescence assays were performed using the H4 antibody (red) that binds all dystrophin-gene products and the 5F3 antibody (green) directed against Dp71 isoforms lacking exon 78. Representative images of cultured neurons from WT (A, B) and KO mice (C, D). Both antibodies labeled neurites and perinuclear regions of control neurons. H4 immunolabeling was present in both control and Dp71-deficient cells, reflecting its binding to several dystrophin-gene products. In contrast, 5F3 antibody labeled neurites of control neurons, but not that of Dp71-deficient neurons. This staining pattern confirms that 5F3 detects specifically Dp71 isoforms lacking exon 78 and expressed in neurites. 5F3 also revealed a basal discontinuous granular labeling in the Golgi complex in both control and Dp71-deficient neurons, which may reflect binding to another dystrophin-like protein in the perinuclear region (Chávez et al., Biochem Biophys Res Commun 274:275–280, 2000). Nuclei stained with DAPI (blue).
(0.66 MB TIF)

Figure S5 Gross hippocampal anatomy is normal in Dp71-null mice. Formalin-fixed brain sections of WT and KO mice were immunostained with various cell markers: NeuN antibody was used to stain neuronal nuclei in CA1-4 and DG regions. Tuj-1 antibody immunostained pyramidal neurons and basal dentrites in CA1. The anti-parvalbumin antibody was used to reveal parvalbumin-containing interneurons.
(3.70 MB TIF)

Figure S6 Basal neurotransmission and PTP in Dp71-null and WT mice. (A–B) Basal glutamatergic transmission mediated through AMPAr: (A) presynaptic fibre volley (PFV) slopes plotted against intensity; (B) AMPAr-mediated fEPSP slopes plotted against intensity. (C–D). NMDAr component of glutamatergic transmission: (C) PFV and (D) NMDAr-mediated fEPSP against intensity. WT(open symbols) and KO mice (black symbols). (E) Time-course of the post-tetanic potentiation (PTP) induced by a 2×100 Hz HFS delivered in the presence of the NMDAr antagonist APV (80 µM). Genotype effect: p>0.37, NS.
(0.62 MB TIF)

Figure S7 Spatial learning and reversal in the water maze. In this experiment, the pool diameter was 1.30 m and mice were submitted to 5 daily trials (Dark symbols, 10 KO, Open symbols, 16 WT). Acquisition was followed by a reversal task (arrow) on day 5. Escape latencies show delayed learning in KO mice during the first two days (p<0.05), with improved performance by day 3–4 and intact reversal.
(0.43 MB TIF)

Table S1 Primary antibodies used in this study. *It could be the immunogen.
(0.05 MB DOC)

Materials and Methods S1 Supplemental Materials and Methods.
(0.04 MB DOC)
