# Correction: Role of Mental Retardation-Associated Dystrophin-Gene Product Dp71 in Excitatory Synapse Organization, Synaptic Plasticity and Behavioral Functions

**DOI:** 10.1371/annotation/1cf456c8-c517-4f04-9e98-8f3ee3b29ce3

**Published:** 2010-01-05

**Authors:** Fatma Daoud, Aurora Candelario-Martínez, Jean-Marie Billard, Avi Avital, Malik Khelfaoui, Yael Rozenvald, Maryvonne Guegan, Dominique Mornet, Danielle Jaillard, Uri Nudel, Jamel Chelly, Dalila Martínez-Rojas, Serge Laroche, David Yaffe, Cyrille Vaillend

This article was originally published with an incorrect publication date. The correct publication date is August 10, 2009. The citation will change to: Daoud F, Candelario-Martínez A, Billard J-M, Avital A, Khelfaoui M, et al. (2009) Role of Mental Retardation-Associated Dystrophin-Gene Product Dp71 in Excitatory Synapse Organization, Synaptic Plasticity and Behavioral Functions. PLoS ONE 4(8): e6574. doi:10.1371/journal.pone.0006574

