# Supplementary figures and images for: Role of Mental Retardation-Associated Dystrophin-Gene Product Dp71 in Excitatory Synapse Organization, Synaptic Plasticity and Behavioral Functions
Source: PLoS One. 2009 Aug 10;4(8):e6574. doi: 10.1371/journal.pone.0006574 (PMC2718704; doi:10.1371/journal.pone.0006574)

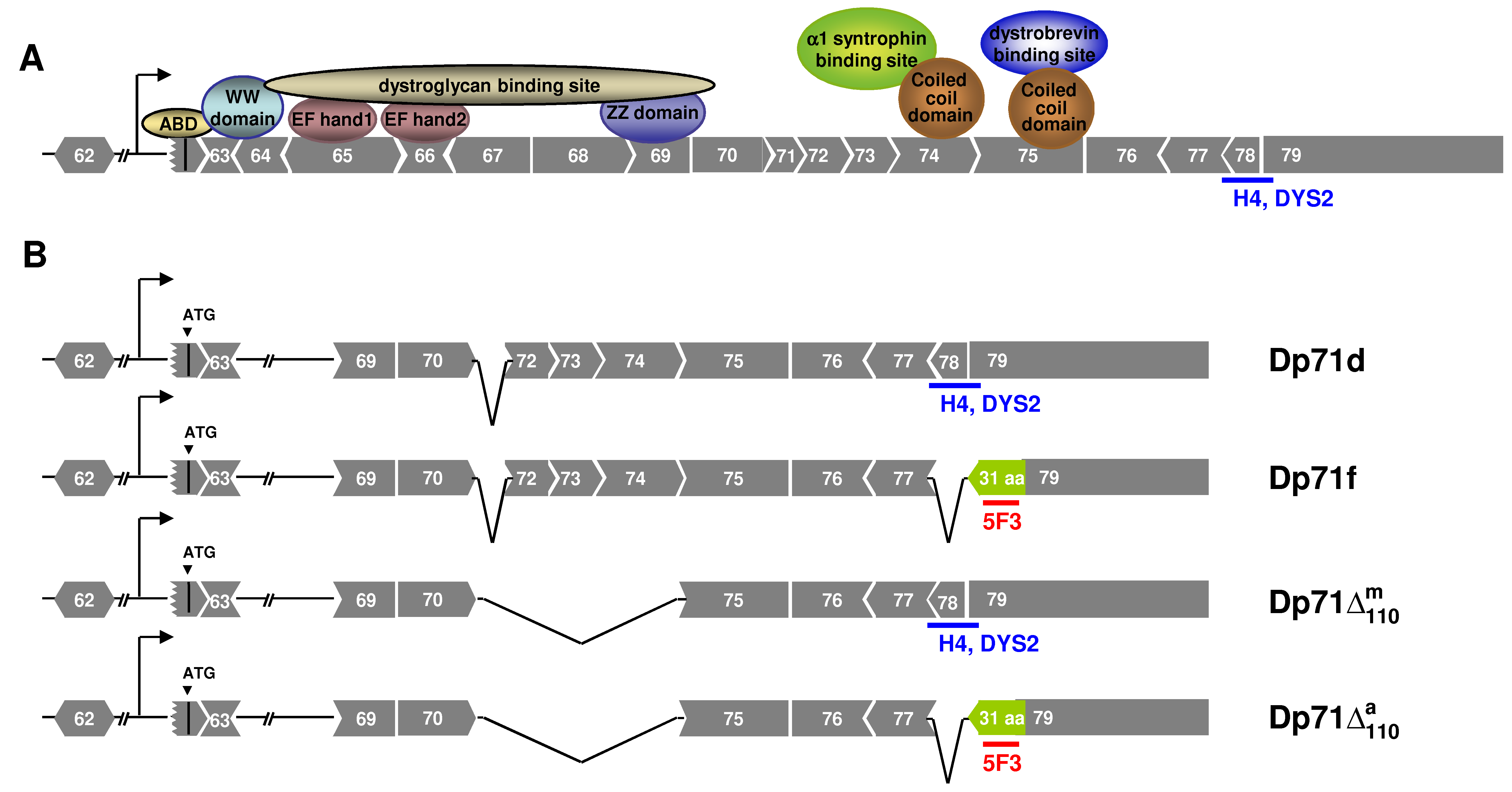

Supplement: Figure S1 — Schematic representation of Dp71 isoforms. (A) Dp71 is transcribed from a promoter located between exons 62 and 63 of the DMD gene; it has a unique N-terminus of seven amino acids and contains the cysteine-rich and common C-terminal domain of dystrophins, a region for binding to DAPs complexes. (B) Dp71 alternative spliced transcripts. Dp71d isoform lacks exon 71 and Dp71f isoform lacks exons 71 and 78 (Marquez et al., Neuroscience 118:957–966, 2003). Deletion of exon 78 changes the reading frame, resulting in the replacement of the last 13 hydrophilic amino acids of dystrophin with 31 new hydrophobic amino acids in the Dp71 protein. Variants of Dp71 protein with minor expression in brain have also been described (Austin et al., Neuromuscul Disord 10(3):187–93, 2000): Dp71Δ110m is derived from Dp71 transcripts deleted for exons 71–74 and contains the C-terminal sequence of muscle dystrophin, while Dp71Δ110a contains the alternative 31-amino acid C-terminal sequence due to the splicing of exon 78 (Austin et al., J Biol Chem 277:47106–47113, 2002). The location of the epitopes recognized by antibodies H4, DYS2 and 5F3 are indicated. (1.01 MB TIF) [file pone.0006574.s001.tif]

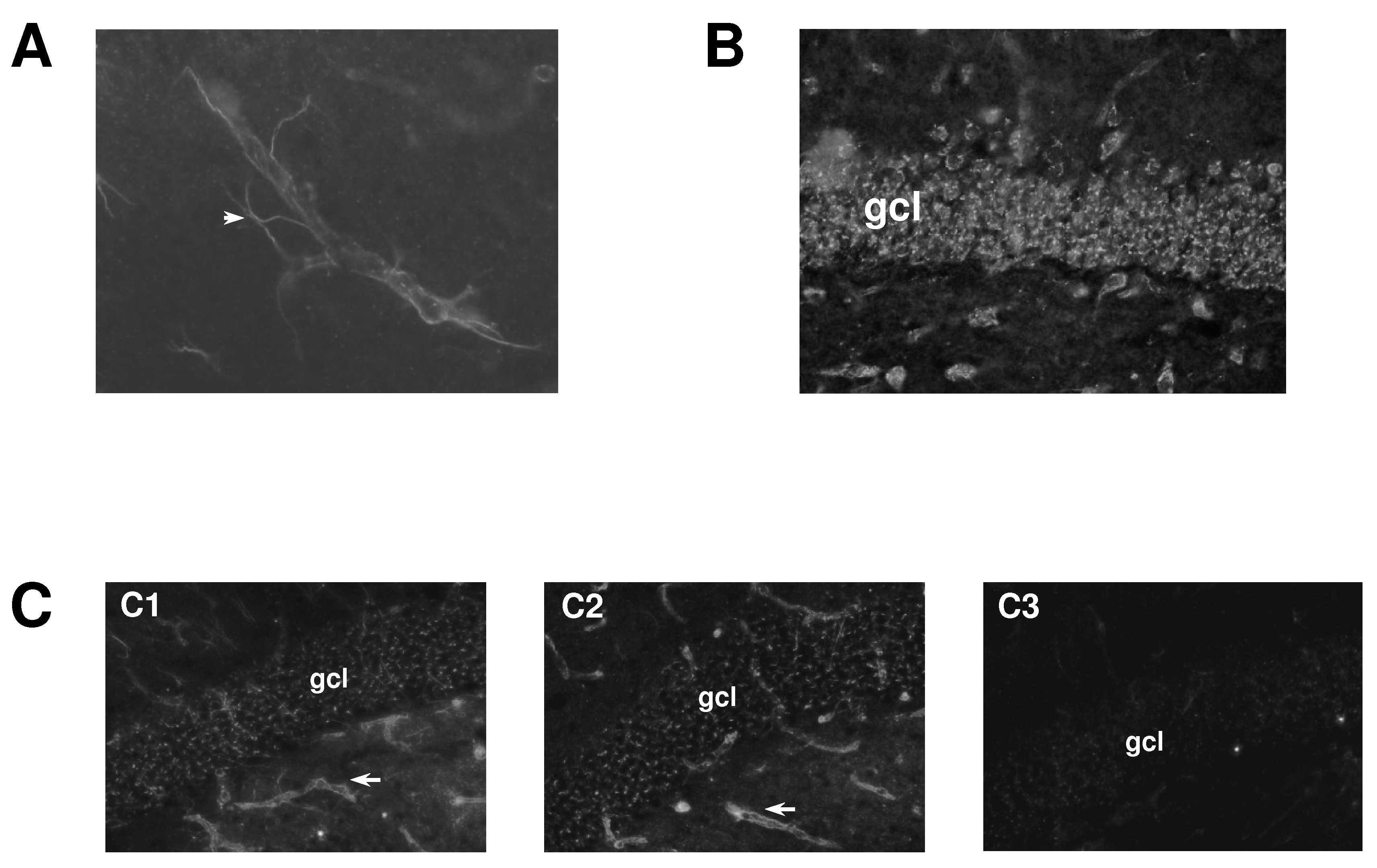

Supplement: Figure S2 — Immunofluorescence detection of Dp71 in brain sections. Hippocampal expression of Dp71 isoforms revealed by H4 on rat brain sections. Representative images show Dp71 expression in (A) walls of blood vessels and perivascular astrocytes (arrowhead), and (B) in the granule-cell layer (gcl) of dentate gyrus as incomplete rings of immunoreactivity circling granule cell bodies and small to large dots around or within cell bodies. C. Immunolabeling of Dp71 with H4 antibody in the gcl and walls of blood vessels (arrowheads) in WT (C1), mdx (C2) and Dp71-null mice (C3). (2.61 MB TIF) [file pone.0006574.s002.tif]

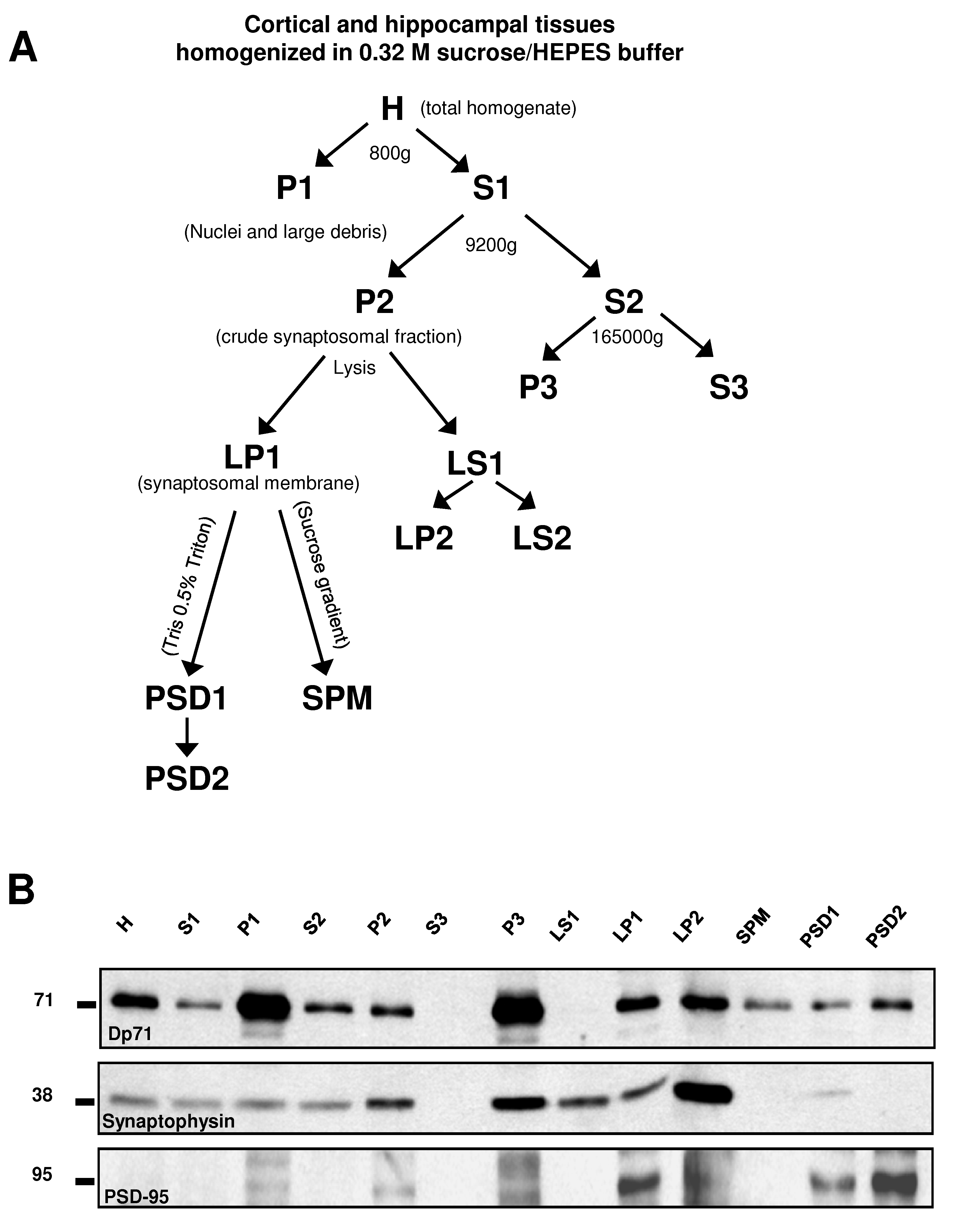

Supplement: Figure S3 — Fractionation study. A. Schematic representation of the fractionation protocol (detailed description in Materials and Methods S1). B. Protein extracts from subcellular fractions obtained from control mouse brains probed with the anti-Dp71 (H4) antibody (top panel). Bottom panels show expression of the presynaptic and postsynaptic markers synaptophysin and PSD-95, respectively. Fractions as follows: H, total homogenate; S1, cell soma; P1, dense nuclei-associated membrane; S2, supernatant, P2: crude synaptosomal membrane, S3, cytosolic; P3, light membrane compartment; LS1, soluble; LP1, synaptosomal membrane; LP2, synaptic-vesicle enriched; SPM, synaptic plasma membrane; PSD1/PSD2, postsynaptic density fractions. (1.84 MB TIF) [file pone.0006574.s003.tif]

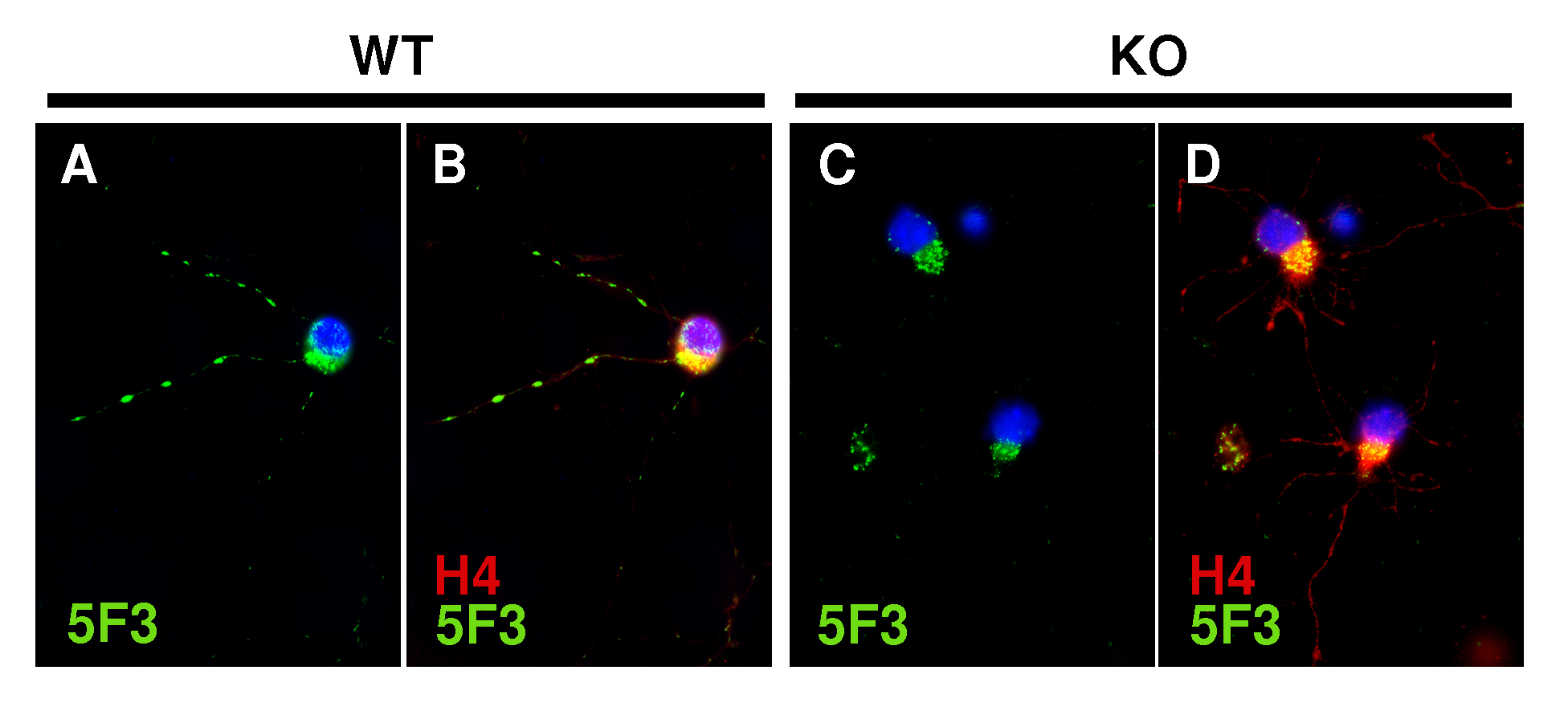

Supplement: Figure S4 — Expression of Dp71 in cultured neurons of control (WT) and Dp71-null (KO) mice. Immunofluorescence assays were performed using the H4 antibody (red) that binds all dystrophin-gene products and the 5F3 antibody (green) directed against Dp71 isoforms lacking exon 78. Representative images of cultured neurons from WT (A, B) and KO mice (C, D). Both antibodies labeled neurites and perinuclear regions of control neurons. H4 immunolabeling was present in both control and Dp71-deficient cells, reflecting its binding to several dystrophin-gene products. In contrast, 5F3 antibody labeled neurites of control neurons, but not that of Dp71-deficient neurons. This staining pattern confirms that 5F3 detects specifically Dp71 isoforms lacking exon 78 and expressed in neurites. 5F3 also revealed a basal discontinuous granular labeling in the Golgi complex in both control and Dp71-deficient neurons, which may reflect binding to another dystrophin-like protein in the perinuclear region (Chávez et al., Biochem Biophys Res Commun 274:275–280, 2000). Nuclei stained with DAPI (blue). (0.66 MB TIF) [file pone.0006574.s004.tif]

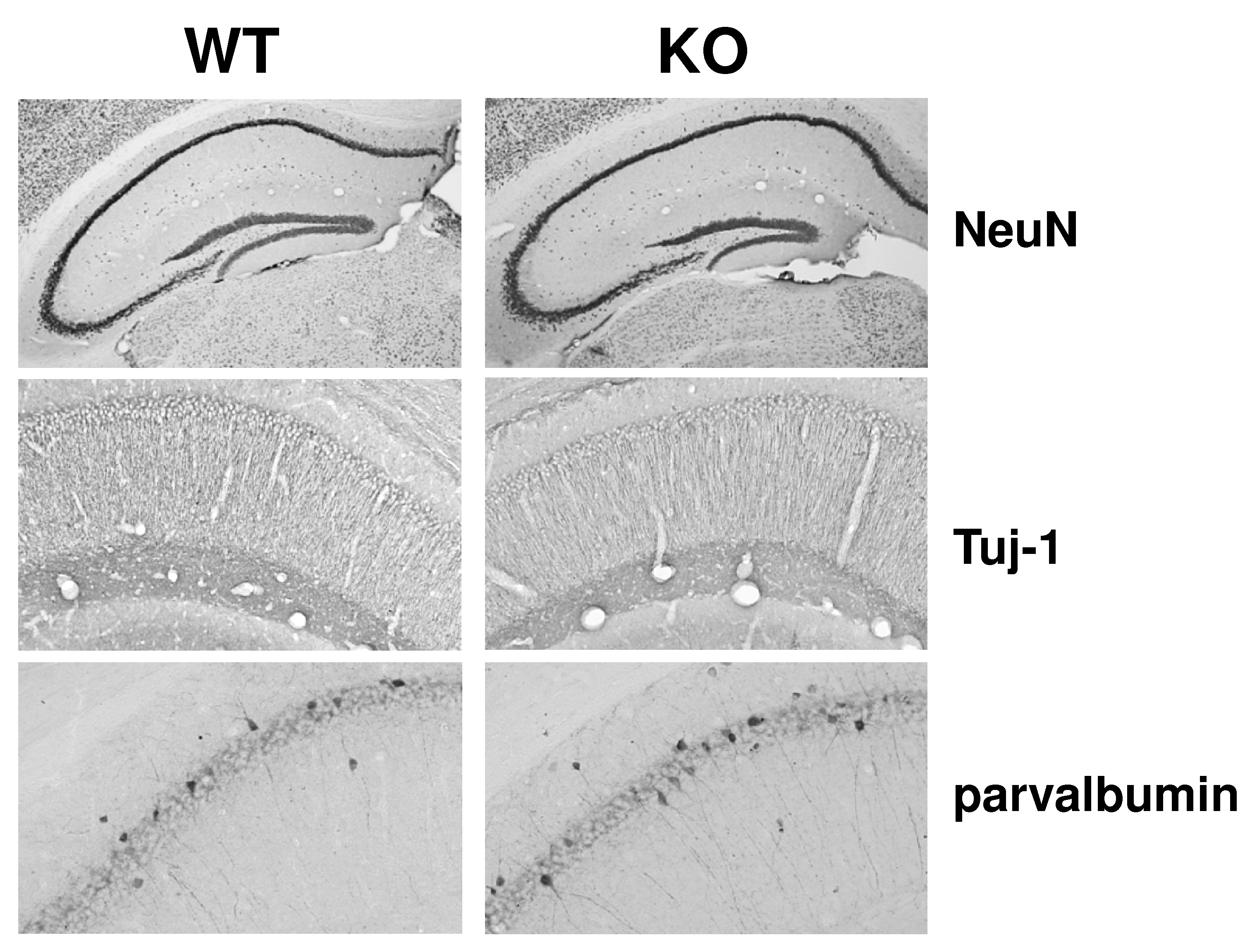

Supplement: Figure S5 — Gross hippocampal anatomy is normal in Dp71-null mice. Formalin-fixed brain sections of WT and KO mice were immunostained with various cell markers: NeuN antibody was used to stain neuronal nuclei in CA1-4 and DG regions. Tuj-1 antibody immunostained pyramidal neurons and basal dentrites in CA1. The anti-parvalbumin antibody was used to reveal parvalbumin-containing interneurons. (3.70 MB TIF) [file pone.0006574.s005.tif]

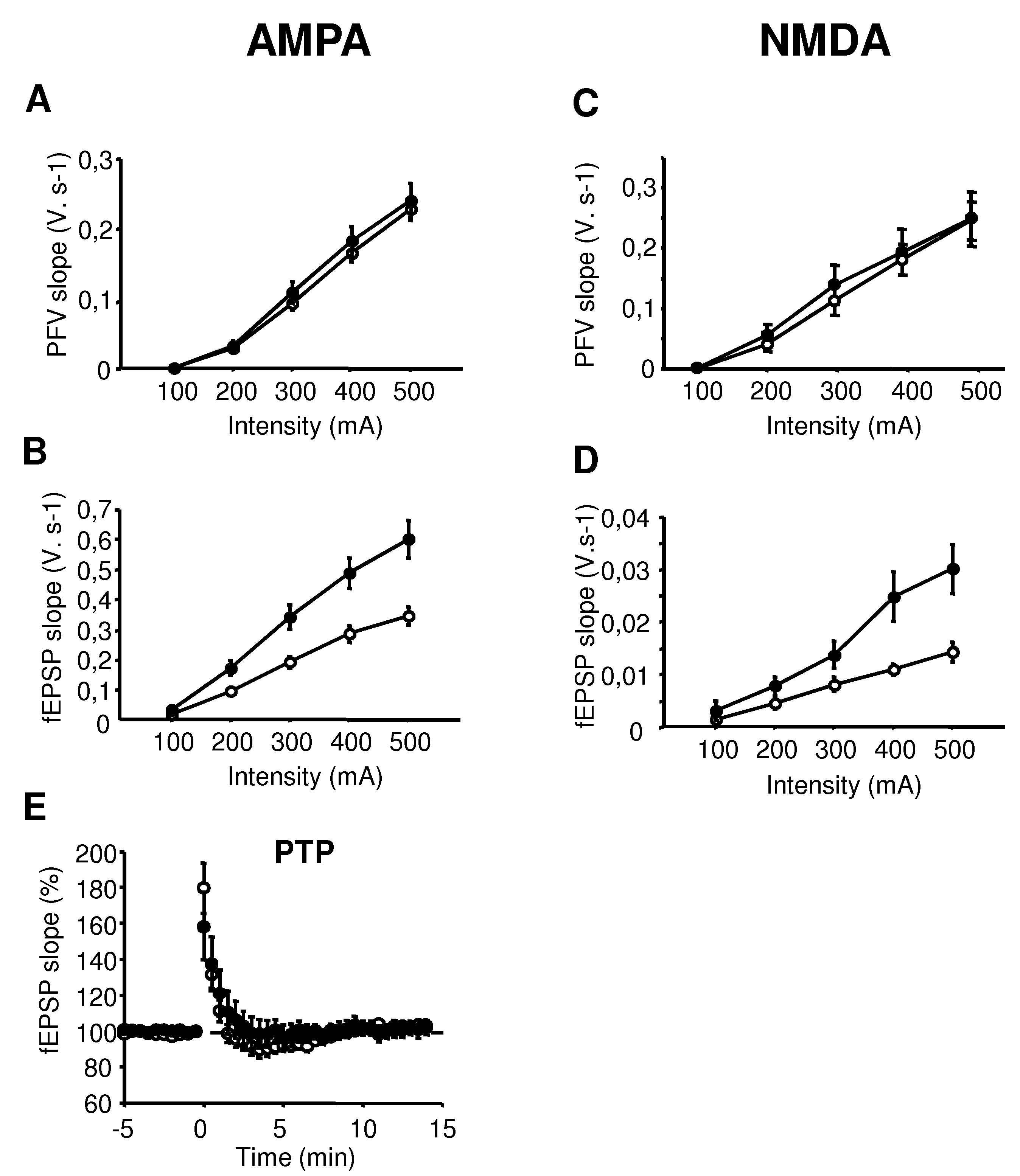

Supplement: Figure S6 — Basal neurotransmission and PTP in Dp71-null and WT mice. (A–B) Basal glutamatergic transmission mediated through AMPAr: (A) presynaptic fibre volley (PFV) slopes plotted against intensity; (B) AMPAr-mediated fEPSP slopes plotted against intensity. (C–D). NMDAr component of glutamatergic transmission: (C) PFV and (D) NMDAr-mediated fEPSP against intensity. WT(open symbols) and KO mice (black symbols). (E) Time-course of the post-tetanic potentiation (PTP) induced by a 2×100 Hz HFS delivered in the presence of the NMDAr antagonist APV (80 µM). Genotype effect: p>0.37, NS. (0.62 MB TIF) [file pone.0006574.s006.tif]

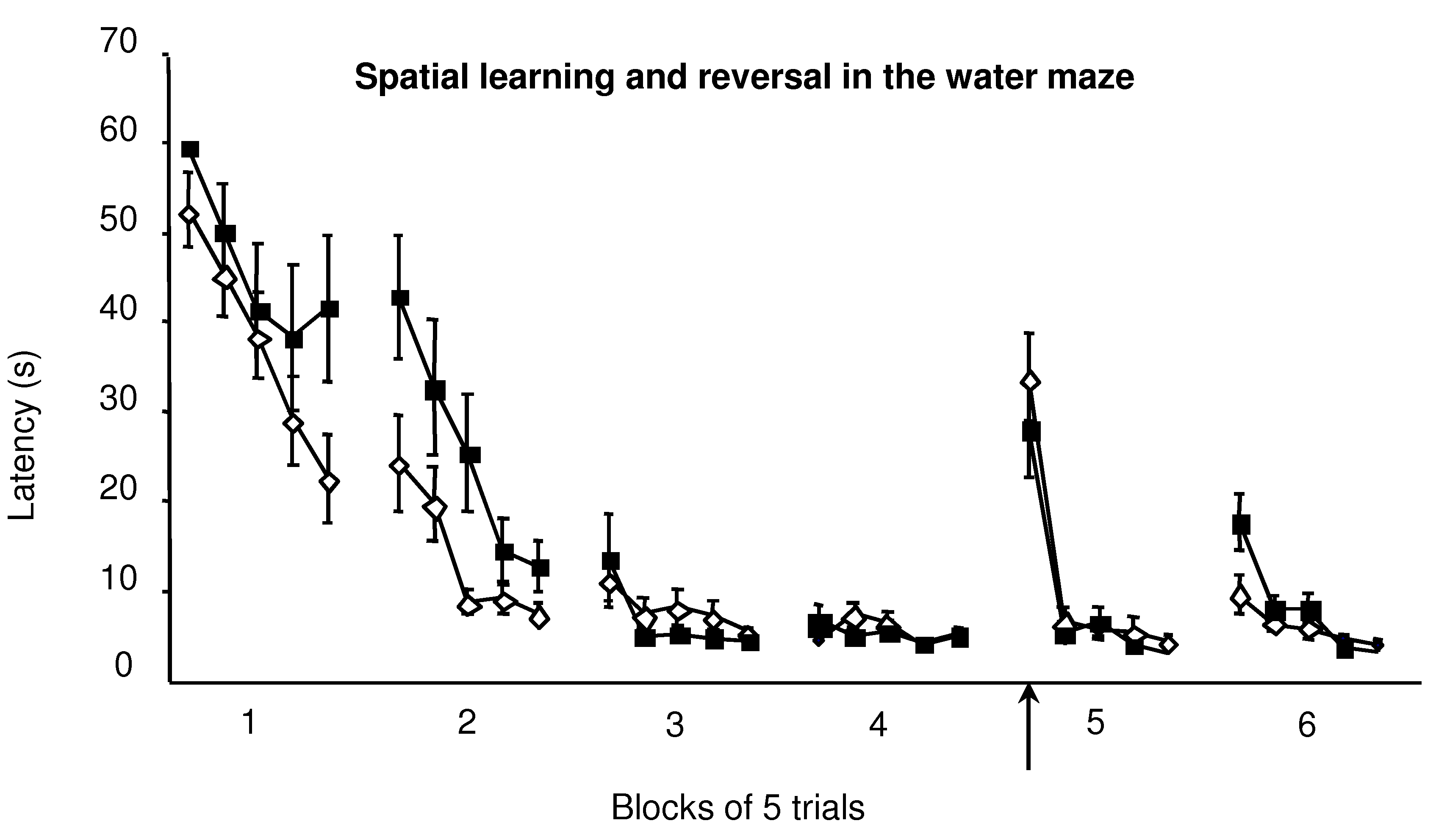

Supplement: Figure S7 — Spatial learning and reversal in the water maze. In this experiment, the pool diameter was 1.30 m and mice were submitted to 5 daily trials (Dark symbols, 10 KO, Open symbols, 16 WT). Acquisition was followed by a reversal task (arrow) on day 5. Escape latencies show delayed learning in KO mice during the first two days (p<0.05), with improved performance by day 3–4 and intact reversal. (0.43 MB TIF) [file pone.0006574.s007.tif]
