# Supplementary material for: Role of Mental Retardation-Associated Dystrophin-Gene Product Dp71 in Excitatory Synapse Organization, Synaptic Plasticity and Behavioral Functions
Source: PLoS One. 2009 Aug 10;4(8):e6574. doi: 10.1371/journal.pone.0006574 (PMC2718704; doi:10.1371/journal.pone.0006574)
Supplement: Table S1 — Primary antibodies used in this study. *It could be the immunogen. (0.05 MB DOC) [file pone.0006574.s008.doc]

| **Table S1.** Primary antibodies used in this study. | | | | |
| --- | --- | --- | --- | --- |
| Specificity | Antibody | Nature | Antigen position* | Reference/Source |
| All dystrophins | H4 | rabbit | C-ter 3663-3678 aa | Dalloz et al., 2003 |
| β-dystroglycan | β-DG | rabbit | C-ter, last 7 aa | Rivier et al.,1999 |
| α-syntrophins | α-Syn | rabbit | 141-206 aa | Rivier et al., 1999 |
| γ1-syntrophin | γ1-Syn | rabbit | C-ter, last 10 aa | Acosta et al., 2004 |
| GAD-65/67 | GAD-65/67 | rabbit |  | B. Giros |
| VGLUT1 | VGLUT1 | rabbit |  | B. Giros |
| GIRK1 | GIRK1 | rabbit | C-ter 480-501 aa | A. Ponce (Ponce et al., 1996) |
| SHANK | SHANK | rabbit |  | M. Sheng |
| FAK | FAK (C20) | rabbit | C-ter of human focal adhesion kinase | Santa Cruz Biotech. |
| PI3 kinase | PI 3-kinase p85 | rabbit | NH2 of the p85 subunit of PI 3 kinase | Santa Cruz Biotech. |
| C-Src | c-Src (N-16) | rabbit | NH2 of s-Src p60 | Santa Cruz Biotech. |
| NMDAr, NR2A-B subunits | NMDAR2A&B | rabbit | LNSCNRRVYKKMPSIESDV | Chemicon Int. |
| All dystrophins | Dys2 | mouse | C-ter, last 17 aa | Novocastra Laboratories |
| Dp71f | 5F3 | mouse | C-ter, founder sequence of Dp71f, last 31 residues | Fabbrizio et al., 1993; 1994 |
| α1,2-dystrobrevins | 610766 | mouse | 249-403 aa | BD Transduction Labs. |
| nNOS | NNOS/NOS | mouse | C-ter 1095-1289 aa | Transduction Labs. |
| α- and β-actin | actin | mouse | Conserved-central region | M. Hernandez  (Díaz-Barriga et al., 1989) |
| Neuron-specific Class III β-tubulin | Tuj-1 | mouse |  | BabCo |
| GLU-R1-3 | Glu 1,2 and 3 | mouse | Rat Glu R fusion proteins | Zymed Laboratories Inc. |
| PSD-95 | PSD95 (6G6) | mouse | Recombinant rat PSD95 | Santa Cruz Biotechnology, Inc. |
| Grb2 | GRB2 | mouse | C-ter 148-217 aa | Santa Cruz Biotech. |
| Neuron-Specific Nuclear Protein | NeuN (A60) | mouse |  | Chemicon |

*It could be the immunogen.

Acosta R, Montanez C, Fuentes-Mera L, Gonzalez E, Gomez P, Quintero-Mora L, Mornet D, Alvarez-Salas LM, Cisneros B (2004) Dystrophin Dp71 is required for neurite outgrowth in PC12 cells. Exp Cell Res 296(2):265-75.

Dalloz C, Sarig R, Fort P, Yaffe D, Bordais A, Pannicke T, Grosche J, Mornet D, Reichenbach A, Sahel J, Nudel U, Rendon A (2003) Targeted inactivation of dystrophin gene product Dp71: phenotypic impact in mouse retina. Hum Mol Genet 12:1543-54.

Díaz-Barriga F, Carrizales L, Yanez L, Hernandez JM, Dominguez RMC, Palmer E, Saborio JL (1989) Interaction of Cadmium with actin microfilaments in vitro. Toxicol in Vitro 3:277-284.

Ponce A, Bueno E, Kentros C, Vega-Saenz de Miera E, Chow A, Hillman D, Chen S, Zhu L, Wu MB, Wu X, Rudy B, Thornhill WB (1996) G-protein-gated inward rectifier K+ channel proteins (GIRK1) are present in the soma and dendrites as well as in nerve terminals of specific neurons in the brain. J Neurosci 16:1990-2001.

Rivier F, Robert A, Hugon G, Bonet-Kerrache A, Nigro V, Fehrentz JA, Martinez J, Mornet D (1999) Dystrophin and utrophin complexed with different associated proteins in cardiac Purkinje fibres. Histochem J 31(7):425-32.
