# Supplementary material for: Role of Mental Retardation-Associated Dystrophin-Gene Product Dp71 in Excitatory Synapse Organization, Synaptic Plasticity and Behavioral Functions
Source: PLoS One. 2009 Aug 10;4(8):e6574. doi: 10.1371/journal.pone.0006574 (PMC2718704; doi:10.1371/journal.pone.0006574)
Supplement: Materials and Methods S1 — Supplemental Materials and Methods. (0.04 MB DOC) [file pone.0006574.s009.doc]

**Supplemental Materials and Methods S1.**

**Fractionation study.**

Brains were dissected on ice. Whole cortical and hippocampal tissues were used to obtain sufficient amount of proteins in the end fractions. All subsequent steps were done at 0-4°C (see Figure S3). Brain tissue was homogenized in ice-cold sucrose/HEPES buffer (0.32M sucrose, 5 mM HEPES, pH 7.4) containing a cocktail of protease inhibitors (Protease inhibitor cocktail tablets, Roche, Germany). Homogenates (H) were centrifuged at 800g for 7 min to remove nuclei and other large debris (pelleted in P1). The supernatant (S1) was centrifuged at 9,200g for 10 min to obtain a crude synaptosomal-membrane fraction (P2). The supernatant (S2) was centrifuged at 165,000g to obtain a cytosolic fraction (S3) and a light membrane fraction (P3). The P2 fraction was lysed hypo-osmotically and centrifuged for 11 min at 25,000g to pellet a synaptosomal membrane fraction (LP1) and supernatant (LS1). The LS1 supernatant was centrifuged at 165,000g to obtain a crude synaptic vesicle-enriched fraction (LP2) and the synaptosomal cytosolic fraction (LS2). One half of the LP1 pellet was resuspended in three volumes of a sucrose/HEPES buffer containing protease inhibitors and was further fractionated on a three-step sucrose gradient by centrifugation at 150,000g for 2h. The fraction from the 1.0/1.2 M interface was recovered and solubilized in 2.5 volumes of ice-cold sucrose/HEPES buffer and then centrifuged at 150,000g for 30 min to obtain the synaptic plasma-membrane fraction (SPM). The other half of LP1 pellet was resuspended in Tris buffer with 0.5% Triton X-100 for 15 min and then centrifuged at 32,000g for 20 min to obtain the first postsynaptic-density pellet (PSD1). The PSD2 fraction was obtained by treating aliquots of PSD1 fraction with 0.5% Triton X-100 in 50 mM Tris buffer (pH 7.4). The suspension was then centrifuged at 200,000g for 1h. PSD1 and PSD2 were resuspended in 50mM Tris-HCl at pH 8.0.

# Electron Microscopy

Mice were perfused with a fixative solution containing 2% paraformaldehyde, 2% glutaraldehyde, 0.002% CaCl_2_ and 0.15% picric acid in 0.1M cacodylate buffer (pH 7.3-7.4). Thick Brain slabs (3mm-thick) containing the whole hippocampus were post-fixed in the same solution overnight at 4ºC. Vibratome sections (250μm-thick) made in the coronal plane were photographed and hippocampal cross-sectional areas were outlined and converted into volume estimation determined as the sum of the traced areas multiplied by the distance between sampled sections. The anterodorsal hippocampus was then dissected under stereomicroscope. Tissue samples were osmicated in 1% buffered osmium tetroxide, soaked in 1% osmium tetroxide and potassium ferrocyanide (10mg/ml) in 0.1M cacodylate buffer, rinsed in buffer and water, contrasted in aqueous solution of uranyl acetate (1%) overnight at 4ºC, dehydrated in graded ethanols, flat-embedded in epon 812 (embed-812, EMS Co. Ltd, Washington, USA) and polymerized at 60º for 48h. Resin blocks were pseudo-randomly selected within a given range of coordinates (bregma -1.70 to -2.04). They were trimmed to a trapezoidal-like region (~1mm^2^) containing the CA1 pyramidal-cell bodies and the entire apical dendritic field of CA1. Ultrathin sections (70-nm thick) of silver-gray interference color were collected on 2x1mm slot grids coated with a formvar-carbon support film and counterstained with uranyl acetate and lead citrate.

## Behavioral testing

*Motor function, exploration and emotional reactivity.* Sensorimotor abilities and muscle strength were evaluated using the inverted grid and wire suspension tests. Locomotion and exploratory behavior were evaluated in the open-field. In the free-choice paradigm, mice were placed in a separate box (20x10 cm) before entering the open-field (110x110 cm, floor divided into virtual sectors of 5x5 grid). After the door was opened, the latency to enter the open-field was recorded and mice were given 5 min of free exploration. Locomotor activity was expressed as the number of sectors crossed in the central area and along the walls while moving in the box (horizontal activity). Time spent in inner squares was recorded as a relative measure of anxiety. In the forced-exploration paradigm, mouse behavior was analyzed during 50 min in 5-min periods every 10 min. Horizontal and vertical (rearing) activities were measured. Emotional reactivity was evaluated with the elevated plus-maze anxiety test as in Poirier et al.[72]. The number of entries and the time spent in open or enclosed arms was recorded for 10 min.

*Spontaneous alternation behavior*. Mice were allowed to freely explore the 4 arms of the maze for 10 min. The number and the temporal order of arm visits were analyzed to quantify alternation rates. Chaining responses (visiting 3 or 4 arms in clockwise or anti-clockwise order) were analyzed as indicators of stereotyped response patterns that may interfere with performance.

*Delayed alternation in a T-maze.* Mice only had access to one lateral alley of a T-maze during 2 successive acquisition trials (ITI: 30s), and they underwent a single retention trial during which the left and right alleys were open for choice after a variable delay (1, 6 or 24h). Mice were submitted to 3 successive acquisition/retention phases at 1w interval.

*Object discrimination in an open-field*. Mice were allowed to freely explore two objects for 10 min during acquisition. Memory retention (5 min) was tested after a delay of 10min, 24 or 48h, by replacing one object by a novel one. Mice were thus submitted to 3 successive acquisition/retention phases with different sets of objects. The latency of the first contact with an object and the time spent in contact with it were recorded. Retention was expressed as the percent time spent exploring the novel object over total object exploration time and was compared to chance (50%).

*Contextual and auditory-cued fear conditioning*. During acquisition, a tone (1000Hz, 80dB, 30s) was delivered as the CS, immediately followed by a footshock (0.3mA, 2s) as the US. Freezing was recorded during 2-min periods before and after the footshocks (5s sampling interval). Retention of conditioned fear was measured 24h later, first by placing the mouse in the same context for 5 min (contextual fear). Three hours later mice were placed in a novel empty box (new context); freezing was recorded for 3 min in this context and for 3 min after turning on the auditory CS (cued-fear).

*Inhibitory avoidance learning*. The apparatus consisted of a dark box (20x10x6 cm) with electrified grid-floor, connected to a brightly lit Plexiglas box (20x10x6 cm) as the shock-free zone. On day 1, mice were submitted to 5 successive acquisition trials. On each trial, mice were placed in the center of the shock-free zone, allowed to explore the apparatus for 10 min, and received a footshock as it stepped into the dark box (0.8mA, 3s). The latency to enter the electrified zone was recorded. In the test session 24h after training, no footshock was given and the step-through latency (600s ceiling) was taken as a measure of retention.

*Spatial learning in a water-maze.* The maze consisted of a circular tank (150-cm diameter) filled with water (22°C) to 15 cm below the top of the sidewall, made opaque by addition of a white nontoxic paint (Opacifier 631, Morton SA, France). A circular escape platform (10-cm diameter) was placed in the centre of the maze during pre-training or the centre of a quadrant (35 cm from the wall) during training. The platform, placed 0.5 cm below the water surface, was not visible. The maze was placed in a well-lit room (380 lux) containing several extra-maze cues on the walls. A video camera, mounted on the ceiling above the maze to record swim paths, was connected to a computer located in an adjacent room, and animals were videotracked using the Anymaze system (Stoelting, USA). The day before training, mice underwent a habituation session, consisting of 4 trials during which the mouse the mouse was gently guided by hand to the platform and allowed to remain on it for 60 s. During the training phase, mice were given one block of four trials a day for 7 days. On each trial, the mouse was introduced into the maze from three different starting points and allowed to swim freely until it reached the platform. Mice failing to find the platform after 90s were gently guided to it by hand and a maximum escape latency of 90 s was recorded. Mice were allowed to remain 60 s on the platform before the start of the next trial. The data recorded by videotracking were used to reconstruct swim paths and to calculate latency to find the platform, averaged swim speed, acceleration during progression segments (darting behavior), swim path lengths, activity in a 19-cm width virtual corridor set along the wall (thigmotaxis), and 360°-rotation of animal’s body (path tortuosity).

In another experiment, a smaller water tank (1.3 m diameter) filled with water was used, which contained a circular transparent escape platform (12 cm diameter) hidden below water surface. The training procedure consisted of 5 trials per day with a 60s cut-off time and a 15-min ITI. Mice were allowed to remain 15s on the platform at the end of a trial. On days 5-6, a reversal test was performed by shifting the platform position to the opposite quadrant.
